# How to Study the Metabolism of New Psychoactive Substances for the Purpose of Toxicological Screenings—A Follow-Up Study Comparing Pooled Human Liver S9, HepaRG Cells, and Zebrafish Larvae

**DOI:** 10.3389/fchem.2020.00539

**Published:** 2020-07-17

**Authors:** Lea Wagmann, Fabian Frankenfeld, Yu Mi Park, Jennifer Herrmann, Svenja Fischmann, Folker Westphal, Rolf Müller, Veit Flockerzi, Markus R. Meyer

**Affiliations:** ^1^Department of Experimental and Clinical Toxicology, Institute of Experimental and Clinical Pharmacology and Toxicology, Center for Molecular Signaling (PZMS), Saarland University, Homburg, Germany; ^2^Department of Microbial Natural Products (MINS), Helmholtz Institute for Pharmaceutical Research Saarland (HIPS), Saarland University, Saarbrücken, Germany; ^3^Environmental Safety Group, Korea Institute of Science and Technology (KIST) Europe, Saarbrücken, Germany; ^4^German Center for Infection Research (DZIF), Partner Site Hannover–Braunschweig, Saarbrücken, Germany; ^5^State Bureau of Criminal Investigation Schleswig–Holstein, Kiel, Germany; ^6^Department of Experimental and Clinical Pharmacology, Institute of Experimental and Clinical Pharmacology and Toxicology, Center for Molecular Signaling (PZMS), Saarland University, Homburg, Germany

**Keywords:** drugs of abuse, zebrafish, metabolism study, isozyme mapping, LC-HRMS/MS, toxicological screening

## Abstract

The new psychoactive substances (NPS) market continues to be very dynamic. A large number of compounds belonging to diverse chemical groups continue to emerge. This makes their detection in biological samples challenging for clinical and forensic toxicologists. Knowledge of the metabolic fate of NPS is crucial for developing comprehensive screening procedures. As human studies are not feasible due to ethical concerns, the current study aimed to compare the NPS' metabolic pattern in incubations with pooled human liver S9 fraction (pHLS9), human liver HepaRG cells, and zebrafish larvae. The latter model was recently shown to be a promising preclinical surrogate for human hepatic metabolism of a synthetic cannabinoid. However, studies concerning other NPS classes are still missing and therefore an amphetamine-based *N*-methoxybenzyl (NBOMe) compound, a synthetic cathinone, a pyrrolidinophenone analog, a lysergamide, as well as another synthetic cannabinoid were included in the current study. Liquid chromatography coupled to Orbitrap-based high-resolution tandem mass spectrometry was used to analyze metabolic data. Zebrafish larvae were found to produce the highest number of phase I but also phase II metabolites (79 metabolites in total), followed by HepaRG cells (66 metabolites). Incubations with pHLS9 produced the least metabolites (57 metabolites). Furthermore, the involvement of monooxygenases and esterases in the metabolic phase I transformations of 4F-MDMB-BINACA was elucidated using single-enzyme incubations. Several cytochrome P450 (CYP) isozymes were shown to contribute, and CYP3A5 was involved in all CYP-catalyzed reactions, while amide and ester hydrolysis were catalyzed by the human carboxylesterase (hCES) isoforms hCES1b and/or hCES1c. Finally, metabolites were compared to those present in human biosamples if data were available. Overall, the metabolic patterns in HepaRG cells provided the worst overlap with that in human biosamples. Zebrafish larvae experiments agreed best with data found in human plasma and urine analysis. The current study underlines the potential of zebrafish larvae as a tool for elucidating the toxicokinetics of NPS in the future.

## Introduction

New psychoactive substances (NPS) are a global issue posing a remarkable challenge to drug policy and a risk to public health. The number of deaths attributed to NPS dramatically increased during the last years, and most NPS appeared to induce more severe adverse effects than classic drugs such as heroin, cannabis, and amphetamine (Kronstrand et al., 2018). Symptoms associated with NPS intake might range from central nervous system toxicity leading to seizures, acute psychosis, aggression, to cardiotoxicity, and liver toxicity resulting in, e.g., arrhythmias and system failures (Kronstrand et al., 2018). NPS products are easily available, mainly via Internet shops selling new compounds as so-called “legal highs” or “research chemicals” (Brandt et al., 2014). Little or no scientific information are available about the effects of NPS and how best to counteract them. Besides, users are often unaware of the content and the dosage of the psychoactive substances contained in NPS products and potentially exposed to additional serious health risks (UNODC, 2019).

**Figure 1:**
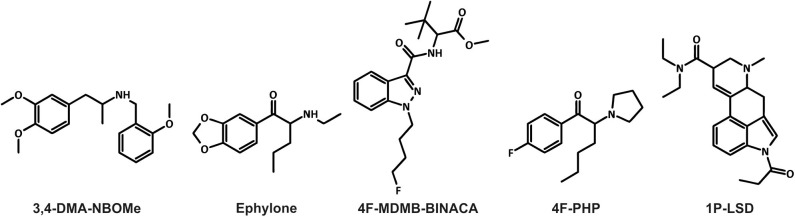
Chemical structures of the five new psychoactive substances included in the current study.

Furthermore, the NPS market is very dynamic and characterized by the large numbers of new substances belonging to several chemical groups (EMCDDA, 2019; UNODC, 2019). Between 2009 and 2016, 739 different NPS were reported to the United Nations Office on Drugs and Crime's Early Warning Advisory (UNODC, 2019). The diversity of compounds available on the market makes the detection of NPS in biological samples challenging for clinical and forensic toxicologists (Wagmann and Maurer, 2018). High-resolution mass spectrometry “is on the way to become the gold standard in non-targeted screening procedures due to its high flexibility, sensitivity, and selectivity” (Maurer and Meyer, 2016; Meyer and Maurer, 2016). The matrix of choice for comprehensive screening purposes is urine, which can be obtained non-invasively, provides large volumes, and the drugs as well as their metabolites are usually concentrated (Peters, 2014). However, lipophilic NPS are often extensively metabolized and the analytical strategy should also be focused on metabolites particularly in urine screening (Wagmann and Maurer, 2018). Thus, metabolism studies are mandatory for developing comprehensive screening methods. Metabolites may be identified in samples obtained from authentic cases. However, authentic human biosamples are often not available and controlled human studies are not feasible due to ethical concerns and lack of preclinical safety data (Meyer, 2016). To overcome this issue, metabolites can be generated using various *in vitro* or *in vivo* models (Meyer, 2018; Wagmann and Maurer, 2018; Diao and Huestis, 2019). Zebrafish (*Danio rerio*) has become a popular model organism. It shares morphological, physiological, and histological characteristics with mammals. For instance, several cytochrome P450 (CYP) enzymes in zebrafish have direct orthologs in humans. It is therefore expected that zebrafish drug metabolism may be similar to that in mammals (De Souza Anselmo et al., 2017). Not only the adult zebrafish, but also zebrafish larvae were used to generate xenobiotic metabolites (Diao and Huestis, 2019). The larvae are not considered as animals until 5 days post-fertilization (dpf) according to the European Directive 2010/63/EU. Despite this, they provide benefits of intact organisms (EU, 2010; Van Wijk et al., 2016). In 2019, Richter et al. used the new synthetic cannabinoid 7′N-5F-ADB (also known as 5F-MDMB-P7AICA and methyl 2-[1-(5-fluoropentyl)-1*H*-pyrrolo[2,3-b]pyridine-3-carboxamido]-3,3-dimethylbutanoate) to further develop the zebrafish larvae model, with the aim to be used as a preclinical surrogate for human hepatic NPS metabolism (Richter et al., 2019a). Results obtained with the larvae model were then compared to those of established *in vitro* incubations using pooled human liver S9 fraction (pHLS9) or the human hepatoma cell line HepaRG. Data of the model systems were also compared to human urinary metabolites (Richter et al., 2019a). Incubations with pHLS9 produced the lowest number of metabolites, while zebrafish larvae and HepaRG cell incubations provided the most comprehensive spectrum of human urinary 7′N-5F-ADB metabolites (Richter et al., 2019a,b). The authors concluded that zebrafish larvae seemed to be a promising model for studying the toxicokinetics of NPS, but further studies comparing different NPS classes were needed (Richter et al., 2019a). Therefore, the aim of the current study was to evaluate five NPS of different classes in a similar manner. Their chemical structures are given in Figure 1. The amphetamine-based NBOMe 3,4-DMA-NBOMe (also known as 3,4-dimethoxyamphetamine-NBOMe and 1-[3,4-dimethoxy]-*N*-[*ortho*-methoxybenzyl]propane-2-amine), the synthetic cathinone ephylone (also known as *N*-ethylnorpentylone and *N*-ethylpentylone), the synthetic cannabinoid 4F-MDMB-BINACA (also known as methyl 2-[1-(4-fluorobutyl)-1*H*-indazole-3-carbonyl]amino-3,3-dimethylbutanoate and 4-fluoro MDMB-BUTINACA), the pyrrolidinophenone analog 4F-PHP (also known as 4-fluoro-alpha-pyrrolidinohexiophenone, 4-fluoro-alpha-pyrrolidinohexanophenone, 4-F-α-PHP, and 4F-alpha-PHP), and the lysergamide 1P-LSD (1-propionyl-*d*-lysergic acid diethylamide) were included. The five NPS were already shown to be extensively metabolized and therefore suitable for the current comparative metabolism study (Caspar et al., 2018b; Haschimi et al., 2019; Krotulski et al., 2019; Wagmann et al., 2019b,c). Caspar et al. used pHLS9 incubations and rat urine to investigate the metabolism of 3,4-DMA-NBOMe (Caspar et al., 2018b; Wagmann et al., 2019b,c). Wagmann et al. identified metabolites of ephylone and 4F-PHP in human blood and urine as well as in pHLS9 incubations (Caspar et al., 2018b; Wagmann et al., 2019b,c). The metabolic fate of 4F-MDMB-BINACA was investigated using human biosamples and incubations with pooled human liver microsomes (pHLM) (Haschimi et al., 2019; Krotulski et al., 2019) and results of *in vitro* incubations with pHLS9 and 1P-LSD were also described (Caspar et al., 2018b; Wagmann et al., 2019b,c).

In the current study, liquid chromatography coupled to Orbitrap-based high-resolution tandem mass spectrometry (LC-HRMS/MS) was used to analyze metabolic data after incubations with zebrafish larvae and HepaRG cells. The metabolites were compared to those detected in pHLS9 incubations, which were already described for four out of five compounds (Caspar et al., 2018b; Wagmann et al., 2019b,c). 4F-MDMB-BINACA pHLS9 incubations should be done in this study. The detected metabolites were compared to those in human biosamples if data were available. To further extend the knowledge about the metabolism of 4F-MDMB-BINACA, monooxygenases and esterases involved in its phase I metabolism should be identified in activity screenings.

## Materials and Methods

### Reagents, Chemicals, and Enzymes

4F-MDMB-BINACA was provided by the EU-funded project ADEBAR (IZ25-5793-2016-27) for research purposes. 3,4-DMA-NBOMe, ephylone, 4F-PHP, and 1P-LSD were available from previous studies (Caspar et al., 2018b; Wagmann et al., 2019b,c). The hydrochloride of racemic 3,4-DMA-NBOMe was provided by the State Bureau of Criminal Investigation Schleswig-Holstein (Kiel, Germany) for research purposes, ephylone and 4F-PHP were obtained from an online vendor of NPS based in the Netherlands, and 1P-LSD was provided by Synex Synthetics (Maastricht, the Netherlands). The identity as well as purity of the NPS were confirmed by high-performance liquid chromatography (HPLC), infrared spectroscopy, and MS/MS. NADP^+^ was obtained from Biomol (Hamburg, Germany), trimipramine-*d*3 was from LGC (Wesel, Germany), and dithiothreitol, reduced glutathione, superoxide dismutase, magnesium chloride (MgCl_2_), dipotassium hydrogen phosphate (K_2_HPO_4_), potassium dihydrogen phosphate (KH_2_PO_4_), Tris hydrochloride, isocitrate dehydrogenase, isocitrate, 3′-phosphoadenosine-5′-phosphosulfate (PAPS), *S*-(5′-adenosyl)-L-methionine (SAM), dimethyl sulfoxide (DMSO), acetyl coenzyme A, diazepam-*d*5, and ammonium formate (analytical grade) were from Merck KGaA (Darmstadt, Germany). Uridine 5′-diphospho-glucuronosyltransferase (UGT) reaction mixture solution A (25 mM UDP glucuronic acid), UGT reaction mixture solution B (250 mM Tris–HCl, 40 mM MgCl_2_, and 0.125 mg/ml alamethicin), pHLS9 (20 mg protein/ml), pHLM (20 mg microsomal protein/ml, 330 pmol total CYP/mg protein), the baculovirus-transfected insect cell microsomes (Supersomes) containing 1 nmol/ml of the human complementary DNA-expressed cytochrome P450 (CYP) isozymes CYP1A2, CYP2A6, CYP2B6, CYP2C8, CYP2C9 (2 nmol/ml), CYP2C19, CYP2D6, CYP2E1 (2 nmol/ml), CYP3A4, CYP3A5 (2 nmol/ml), flavin-containing monooxygenase 3 (FMO 3, 5 mg protein/ml), and recombinant human carboxylesterases hCES1b, hCES1c, and hCES2 were obtained from Corning (Amsterdam, the Netherlands). All enzyme containing preparations were thawed at 37°C after delivery, aliquoted, snap-frozen in liquid nitrogen, and stored at −80°C until use. Williams Medium E, HPRG670 supplement, GlutaMAX, cryopreserved and differentiated HepaRG cells, and 96-well plates coated with type I collagen were obtained from Life Invitrogen (Darmstadt, Germany). Zebrafish embryos were obtained from in-house bred adult zebrafish (approval no. 2.4.1.1-H, Landesamt für Verbraucherschutz, Saarland, Germany) of the AB wild-type line, and 6-well plates and 96-well plates were obtained from Sarstedt (Nümbrecht, Germany). Acetonitrile (LC-MS grade), methanol (LC-MS grade), formic acid (LC-MS grade), and all other reagents and chemicals (analytical grade) were purchased by VWR (Darmstadt, Germany).

### Incubations With pHLS9 and 4F-MDMB-BINACA

According to published procedures (Richter et al., 2017b, 2019b; Caspar et al., 2018b; Wagmann et al., 2019b,c), the final incubation volume per reaction tube was 150 μl, with a final protein concentration of 2 mg/ml. The given concentrations represent final concentrations. A preincubation for 10 min at 37°C with UGT reaction mixture solution B (containing alamethicin, 25 μg/ml), MgCl_2_ (2.5 mM), NADP^+^ (0.6 mM), isocitrate (2.5 mM), isocitrate dehydrogenase (0.8 U/ml), superoxide dismutase (100 U/ml), acetyl coenzyme A (0.1 mM), phosphate buffer (90 mM, pH 7.4), and pHLS9 (2 mg/ml) was done. Afterwards, UGT reaction mixture solution A (containing UDP glucuronic acid, 2.5 mM), PAPS (40 μM), SAM (1.2 mM), dithiothreitol (1 mM), and glutathione (10 mM) were added, as well as 25 μM 4F-MDMB-BINACA to start the reactions. The mixture was incubated for 360 min. Total amount of organic solvent in incubation mixtures was <2% (Chauret et al., 1998). After 60 min, an aliquot of the reaction mixture (60 μl) was transferred in another reaction tube containing 20 μl of ice-cold acetonitrile to terminate the reactions. The remaining aliquot (90 μl) was incubated for an additional 300 min, and then the reactions were stopped by adding 30 μl of ice-cold acetonitrile. Thereafter, the reaction tubes were cooled at −20°C for 30 min prior to centrifugation at 18,407 × *g* for 2 min. The supernatants (50 μl) were transferred into autosampler vials, and 1 μl was injected onto the LC-HRMS/MS system. Control samples without pHLS9 and blank samples without substrate were prepared to confirm the absence of non-metabolically formed and interfering compounds, respectively. All incubations were performed in duplicate.

### *In vivo* Maximum-Tolerated Concentration Studies in Zebrafish Larvae

Internal protocols based on published standard methods (Westerfield, 2007) were used to perform zebrafish husbandry and the maximum-tolerated concentration (MTC) testing was based on a previous study (Richter et al., 2019a). Danieau's medium [17 mM NaCl, 2 mM KCl, 0.12 mM MgSO_4_, 1.8 mM Ca(NO_3_)_2_, 1.5 mM HEPES, pH 7.1–7.3, and 1.2 μM methylene blue] at 28°C was used to raise zebrafish larvae of the AB wild-type strain. For MTC testing, 96-well plates were used with 100 μl of medium and one larva per well. Drug exposition was started 4 dpf using 0, 0.01, 0.1, 1, 10, 50, or 100 μM final concentrations of 3,4-DMA-NBOMe, ephylone, 4F-MDMB-BINACA, 4F-PHP, or 1P-LSD, respectively. The drug exposure in the incubator at 28°C lasted 24 h. The final DMSO concentration was 1% (*v/v*) for all experiments. Monitoring of the larvae was done using a LEICA M205 FA stereo microscope (Leica Mikrosysteme Vertrieb GmbH, Wetzlar, Germany) to detect developmental defects and/or decreased survival rates. Fifteen larvae were used for each experiment.

### Zebrafish Larvae Exposure

In accordance with a previous study (Richter et al., 2019a) with minor modifications, substances were administrated to the zebrafish larvae at 4 dpf via the Danieau's medium with final substrate concentrations of 25 μM (4F-MDMB-BINACA) or 100 μM (3,4-DMA-NBOMe, ephylone, 4F-PHP, and 1P-LSD), respectively, and a final DMSO concentration of 1% (*v/v*). Six-well plates were used and 10 larvae were placed in each well containing 2,000 μl of Danieau's medium supplemented with one of the NPS. After an incubation period of 24 h at 28°C, the larvae were collected separated from the surrounding medium. All larvae of one well (10 larvae) were washed twice with 1,000 μl of Danieau's medium. Afterwards, they were euthanized by placing the tubes in ice water for 15 min. After removing the residual medium, the larvae were snap-frozen in liquid nitrogen followed by lyophilization and stored at −20°C until extraction. Twenty larvae (from two wells) were extracted using 50 μl of methanol, shaken for 2 min, and centrifuged for 2 min at 18,407 × *g*. Thirty microliters of the supernatants was transferred into autosampler vials and 1 μl was injected onto the LC-HRMS/MS system. Medium samples without larvae were collected to identify non-metabolically formed compounds. Blank larvae samples without the NPS were prepared to confirm the absence of interfering compounds. All incubations were done in triplicate.

### Incubations With HepaRG Cells

Cell culture experiments using differentiated human hepatocellular carcinoma HepaRG cells were carried out as recently described (Richter et al., 2019a,c). Cells were handled under sterile conditions. A laminar flow bench class II (Thermo Scientific Schwerte, Germany) and an incubator (Binder, Tuttlingen, Germany) with 95% air humidity and 5% CO_2_ atmosphere at 37°C were used. The concentrations given in the following are final concentrations. Collagen-coated 96-well plates were used and the HepaRG cells were seeded in a density of 72,000 cells/well (a 100-μl aliquot cell suspension/well) in thaw and seed medium, consisting of Williams E medium supplemented with penicillin (100 U/ml), streptomycin (100 μg/ml), GlutaMAX, and HPRG670. Four hours after cell seeding, 50 μl of the supernatants was removed from each well. Afterwards, 50 μl of the thaw and seed medium containing 3,4-DMA-NBOMe, ephylone, 4F-MDMB-BINACA, 4F-PHP, or 1P-LSD (50 or 500 μM resulting in final concentrations of 25 or 250 μM), respectively, was added. Each well also contained 0.5% (*v/v*) DMSO. After drug exposure of 24 h, 50 μl of the supernatants was precipitated using the same volume of acetonitrile containing 0.1% (*v/v*) formic acid. The mixtures were vortexed, cooled for 30 min at −20°C, and centrifuged at 18,407 × *g* for 2 min. Eighty microliters of the mixture was transferred into an autosampler vial and 1 μl was injected onto the LC-HRMS/MS system. Control samples without substrate and blank samples without HepaRG cells were prepared in the same manner to confirm the absence of interfering compounds or non-metabolically formed compounds, respectively. All incubations were performed in triplicate.

### Monooxygenases Activity Screening of 4F-MDMB-BINACA

According to an established protocol (Wagmann et al., 2016), the final volume per reaction tube was 50 μl with a final 4F-MDMB-BINACA concentration of 25 μM and enzyme concentrations of 50 pmol/ml for CYP1A2, CYP2A6, CYP2B6, CYP2C8, CYP2C9, CYP2C19, CYP2D6, CYP2E1, CYP3A4, and CYP3A5, respectively, or 0.25 mg protein/ml for FMO3. Furthermore, the reaction mixture contained 90 mM phosphate buffer (pH 7.4), superoxide dismutase (200 U/ml), isocitrate (5 mM), MgCl_2_ (5 mM), isocitrate dehydrogenase (0.5 U/ml), and NADP^+^ (1.2 mM). The phosphate buffer was replaced with Tris buffer (90 mM) for incubations with CYP2A6 and CYP2C9 according to the manufacturer's recommendation. All incubations were performed at 37°C for 30 min and terminated by adding 50 μl of ice-cold acetonitrile. Afterwards, the mixtures were centrifuged at 18,407 × *g* for 5 min, 70 μl of the supernatants was transferred into autosampler vials, and 1 μl was injected onto the LC-HRMS/MS system. Positive control incubations were performed with pHLM (1 mg microsomal protein/ml), and negative controls without enzyme were prepared to identify non-metabolically formed compounds. All incubations were done in duplicate.

### Esterases Activity Screening of 4F-MDMB-BINACA

Experiments were performed in accordance with a previous study (Meyer et al., 2015) with minor modifications. The final volume per reaction tube was 100 μl with a final 4F-MDMB-BINACA concentration of 100 μM and enzyme concentration of 0.2 mg protein/ml for hCES1b, hCES1c, and hCES2. Incubations with pHLM and pHLS9 (0.2 mg protein/ml, each) were performed in a similar manner. Additionally, incubations with human plasma (50 μl pooled human plasma, 40 μl phosphate buffer, and 10 μl substrate) were performed. The reactions were started by adding the enzyme-containing preparation and terminated with an equal volume of ice-cold acetonitrile containing diazepam-*d*5 (100 μM) as an internal standard. Only plasma incubations were terminated by addition of a three-fold volume of ice-cold acetonitrile. The reaction mixtures were centrifuged at 18,407 × *g* for 15 min, and then supernatants were transferred to autosampler vials, and 5 μl was injected onto the LC-HRMS/MS system. Negative control incubations without enzyme were prepared to identify non-metabolically formed compounds. All incubations were done in duplicate.

### Preparation of Human Biosamples After Intake of 4F-MDMB-BINACA

Human blood and urine collected after a suspected intake of drugs of abuse were submitted to the authors' laboratory for regular clinical toxicological analysis. The blood sample was centrifuged, and plasma was separated. The authors' standard liquid–liquid extraction for human plasma screening was used (Maurer et al., 2016). Briefly, 1,000 μl of plasma was mixed with 100 μl of internal standard (0.01 μg/μl trimipramine-*d*3 in methanol) and extracted with 5,000 μl of a mixture of diethyl ether:ethyl acetate (1:1, *v/v*) after addition of 2,000 μl of saturated sodium sulfate solution. Phase separation was mediated by centrifugation. The organic layer was transferred into a flask and evaporated to dryness at 60°C under reduced pressure. The aqueous residue was then alkalized with 500 μl of 1 M sodium hydroxide and extracted a second time with 5,000 μl of the diethyl ether:ethyl acetate mixture. The organic extract was transferred to the same flask and evaporated as described before. The combined residues were dissolved in 100 μl of methanol and transferred into an autosampler vial, and 5 μl was injected onto the LC-HRMS/MS system. An aliquot of the human urine sample (100 μl) was prepared according to a previous study by precipitation with 500 μl of acetonitrile (Wissenbach et al., 2011). The mixture was shaken and centrifuged (18,407 × *g*, 2 min). After transfer of the supernatant into an autosampler vial, it was evaporated to dryness at 70°C under a gentle stream of nitrogen. The residue was dissolved in 50 μl of a mixture of eluent A and B (see LC-HRMS/MS conditions, 1:1, *v/v*) and 5 μl was injected onto the LC-HRMS/MS system.

### LC-HRMS/MS Apparatus and Conditions

According to a previous study (Richter et al., 2019a), a Thermo Fisher Scientific (TF, Dreieich, Germany) Dionex UltiMate 3000 Rapid Separation (RS) UHPLC system with a quaternary UltiMate 3000 RS pump and an UltiMate 3000 RS autosampler controlled by the TF Chromeleon software version 6.8 was used. The LC system was coupled to an Orbitrap-based TF Q-Exactive Plus equipped with a heated electrospray ionization II (HESI-II) source. Calibration was done prior to analysis with a Positive Cal Mix (Supelco, Bellefonte, PA, USA) at a flow rate of 3 μl/min using a syringe pump. The conditions of the LC system were as follows: TF Accucore PhenylHexyl column (100 × 2.1 mm, 2.6 μm particle size); gradient elution with 2 mM ammonium formate solution containing 0.1% (*v/v*) formic acid (eluent A) and 2 mM ammonium formate solution in acetonitrile/methanol (50:50, *v/v*) containing 0.1% (*v/v*) formic acid, and 1% (*v/v*) water (eluent B). The flow rate was set to 0.5 ml/min (0–11.5 min) and 0.8 ml/min (11.5–13.5 min). The following gradient was used: 0–1.0 min hold 1% B, 1–10 min to 99% B, 10–11.5 min hold 99% B, 11.5–13.5 min hold 1% B. The HESI-II source conditions were as follows: ionization mode, positive; heater temperature, 320°C; ion transfer capillary temperature, 320°C; sheath gas, 60 arbitrary units (AU); auxiliary gas, 10 AU; spray voltage, 4.00 kV; and S-lens RF level, 50.0. Mass spectrometry experiments were performed using high-resolution (HR) full scan mode and a targeted MS^2^ mode with an inclusion list for each NPS. The inclusion list contained *m/z* values of metabolites, which were likely to be formed for example hydroxy, oxo, carboxy, dealkyl, demethylenylated, and defluorinated metabolites (phase I) as well as sulfates and glucuronides (phase II) and combinations of them. Full scan settings were as follows: resolution, 35,000; automatic gain control (AGC) target, 3e6; maximum injection time (IT), 120 ms; scan ranges, 3,4-DMA-NBOMe *m/z* 100–700, ephylone *m/z* 200–600, 4F-MDMB-BINACA *m/z* 150–700, 4F-PHP *m/z* 200–600, and 1P-LSD *m/z* 250–700. The settings for the targeted MS^2^ mode with the respective inclusion lists were as follows: resolution, 17,500; AGC target, 2e5; maximum IT, 250 ms; loop count, 5; isolation window, 1.0 *m/z*; stepped normalized collision energy (NCE), 17.5, 35, 52.5%; pick others, enabled. ChemSketch 2018 2.1 (ACD/Labs, Toronto, Canada) was used to draw the structures of the hypothetical metabolites and for the calculations of the exact masses. Xcalibur Qual Browser version 4.1.31.9 (TF) was used for data handling. The mass tolerance between calculated and measured mass was adjusted to 5 ppm. For identification of metabolites, HR full scan data were screened for potential exact precursor ion (PI) masses of expected metabolites. Metabolites were tentatively identified by comparing the corresponding HRMS^2^ spectrum to that of the parent compound.

## Results and Discussion

### Identification of 4F-MDMB-BINACA Phase I and II Metabolites in pHLS9 Incubations

**Figure 2:**
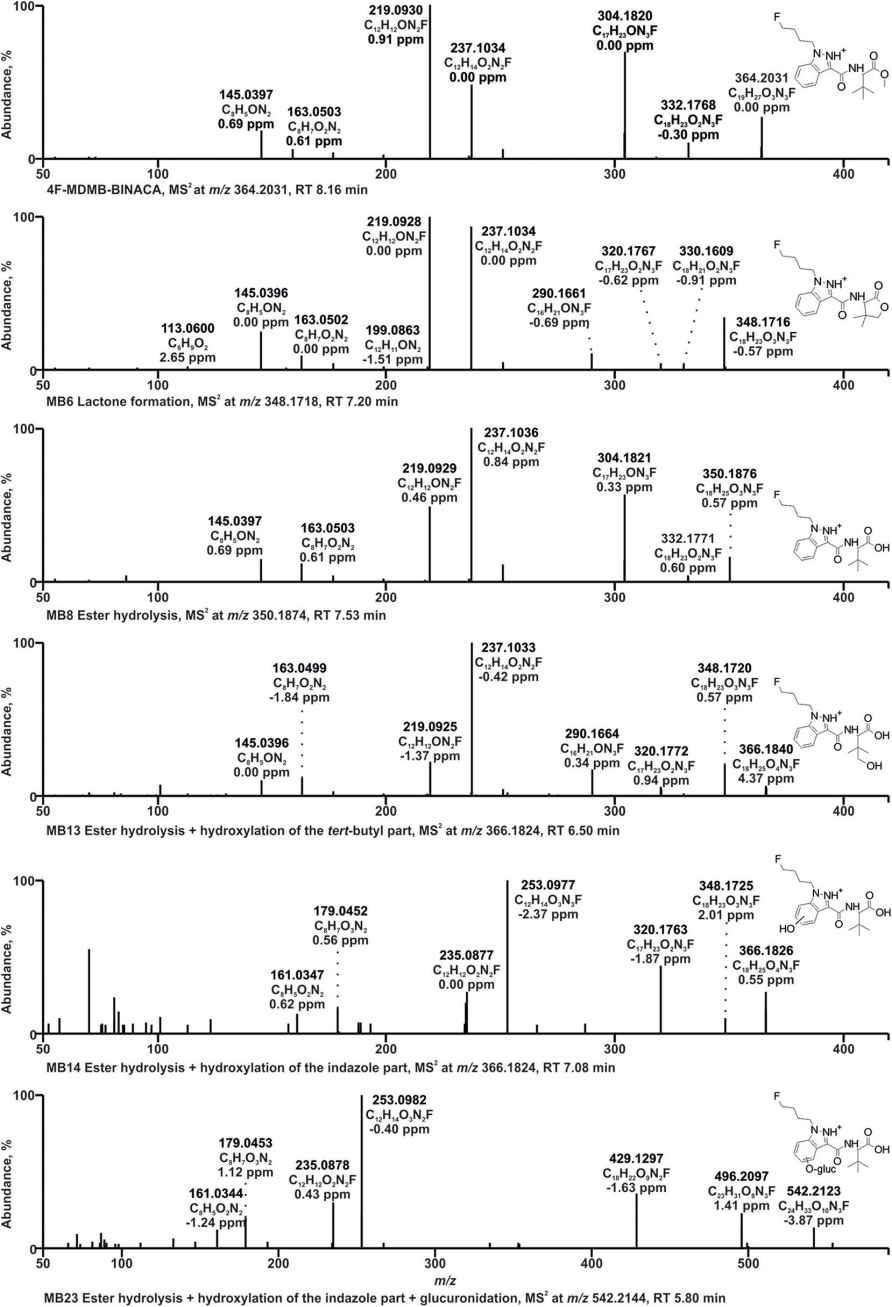
HRMS^2^ spectra of 4F-MDMB-BINACA and some of its metabolites detected in incubations with pooled human liver S9 fraction, HepaRG cells, and/or zebrafish larvae. Metabolite IDs correspond to Table 1. MB, 4F-MDMB-BINACA metabolite; RT, retention time.

Only the reference material of the parent compound 4F-MDMB-BINACA was available. Therefore, postulated metabolic transformations could not be confirmed using chemical standards, and all the metabolites were claimed to be tentatively identified. This was also true for the other investigated NPS. All tentatively identified metabolites of 4F-MDMB-BINACA along with their absolute peak areas in pHLS9 incubations after 1 or 6 h of incubations are listed in Table 1. The given areas were taken from one replicate of the pHLS9 incubations (*n* = 2). The second replicate provided the same pattern of metabolite abundances. HRMS^2^ spectra of the parent compound and the metabolites discussed in detail in the following are depicted in Figure 2, while HRMS^2^ spectra of all other 4F-MDMB-BINACA metabolites are depicted in the Electronic Supplementary Material (ESM). The metabolites were sorted by increasing *m/z*. In case of isomers with identical *m/z*, metabolites were sorted by increasing retention time. Each metabolite was assigned to a unique metabolite ID. The calculated exact masses, not the measured masses, will be used in the following. In total, 15 phase I and two phase II metabolites of 4F-MDMB-BINACA were detected in pHLS9 incubations (see Table 2). Haschimi et al. performed 4F-MDMB-BINACA incubations with pHLM instead of pHLS9 without co-substrates of phase II metabolic reactions and identified 11 phase I metabolites (Haschimi et al., 2019). All these metabolites were also detected in pHLS9 incubations with one exception, namely, a 4F-MDMB-BINACA metabolite formed after ester hydrolysis and hydroxylation of the butyl chain.

**Table 1:** Absolute peak areas of 4F-MDMB-BINACA and its phase I and II metabolites in incubations with pooled human liver S9 fraction (pHLS9, *n* = 2), HepaRG cells (*n* = 3), and zebrafish larvae (*n* = 3) taken from one replicate, each, along with the unique metabolite ID, observed metabolic reaction, and calculated exact mass.

**Metabolite ID**	**Metabolic reaction**	**Calculated exact masses, *m/z***	**pHLS9 1 h [25 μM]**	**pHLS9 6 h [25 μM]**	**HepaRG [25 μM]**	**HepaRG [250 μM]**	**Zebrafish larvae [25 μM]**
4F-MDMB-BINACA	- (parent compound)	364.2031	**9.37E+08**	**2.17E+08**	**1.28E+08**	**5.18E+08**	**2.89E+09**
MB1	Amide hydrolysis	237.1034	n.d.	n.d.	3.34E+04	3.82E+06	n.d.
MB2	Lactone formation + *N*-dealkylation	274.1186	n.d.	n.d.	n.d.	n.d.	5.13E+05
MB3	Ester hydrolysis + *N*-dealkylation	276.1343	9.07E+05	1.27E+06	3.99E+05	6.92E+06	n.d.
MB4	*N*-Dealkylation	290.1499	**5.62E+06**	2.82E+05	n.d.	9.55E+06	7.45E+06
MB5	Lactone formation + oxidative defluorination	346.1761	n.d.	1.26E+06	n.d.	2.40E+05	9.42E+05
MB6	Lactone formation	348.1718	n.d.	**5.21E+07**	n.d.	2.48E+06	**1.29E+08**
MB7	Ester hydrolysis + oxidative defluorination	348.1918	n.d.	**1.91E+06**	**1.25E+06**	**1.34E+07**	5.96E+04
MB8	Ester hydrolysis	350.1874	**1.01E+08**	**2.77E+08**	**5.16E+07**	**1.63E+09**	**1.73E+07**
MB9	Lactone formation + oxidative defluorination + oxidation to carboxylic acid	360.1554	n.d.	1.57E+05	n.d.	n.d.	2.53E+06
MB10	Ester hydrolysis + oxidative defluorination + oxidation to carboxylic acid	362.1710	n.d.	n.d.	1.68E+05	8.32E+05	5.72E+04
MB11	Oxidative defluorination	362.2074	**4.27E+06**	3.89E+05	9.30E+04	6.93E+06	2.92E+06
MB12	Lactone formation + hydroxylation of the *tert*-butyl part	364.1667	n.d.	n.d.	n.d.	n.d.	1.68E+06
MB13	Ester hydrolysis + hydroxylation of the *tert*-butyl part	366.1824	7.30E+03	6.42E+05	n.d.	**3.13E+07**	4.57E+05
MB14	Ester hydrolysis + hydroxylation of the indazole part	366.1824	4.94E+04	3.04E+05	n.d.	n.d.	n.d.
MB15	Oxidative defluorination + oxidation to carboxylic acid	376.1867	2.16E+06	**3.34E+06**	1.15E+04	8.00E+06	**2.60E+07**
MB16	Hydroxylation of the *tert*-butyl part	380.1980	1.70E+05	n.d.	n.d.	n.d.	5.98E+06
MB17	Hydroxylation of the butyl chain	380.1980	9.01E+05	5.84E+04	n.d.	2.95E+06	n.d.
MB18	Hydroxylation of the indazole part isomer 1	380.1980	3.75E+05	n.d.	n.d.	n.d.	4.77E+05
MB19	Hydroxylation of the indazole part isomer 2	380.1980	**7.16E+06**	4.56E+04	n.d.	n.d.	n.d.
MB20	Hydroxylation of the indazole part + sulfation	460.1548	n.d.	n.d.	n.d.	n.d.	2.06E+06
MB21	Ester hydrolysis + oxidative defluorination + glucuronidation	524.2239	n.d.	n.d.	**4.18E+05**	7.56E+05	n.d.
MB22	Ester hydrolysis + glucuronidation	526.2196	n.d.	n.d.	**2.56E+07**	**3.25E+07**	n.d.
MB23	Ester hydrolysis + hydroxylation of the indazole part + glucuronidation	542.2144	n.d.	1.34E+05	n.d.	n.d.	n.d.
MB24	Oxidative defluorination + oxidation to carboxylic acid + glucuronidation	552.2188	n.d.	n.d.	n.d.	n.d.	2.34E+05
MB25	Hydroxylation of the *tert*-butyl part + glucuronidation	556.2301	n.d.	n.d.	n.d.	n.d.	**1.75E+07**
MB26	Hydroxylation of the indazole part + glucuronidation	556.2301	5.96E+05	7.68E+05	n.d.	1.48E+06	1.96E+06

*The five largest areas per model are given in bold. MB, 4F-MDMB-BINACA metabolite; n.d., not detected*.

**Table 2:** Number of phase I and II metabolites of the five tested NPS detected in incubations with pooled human liver S9 fraction (pHLS9), HepaRG cells, and zebrafish larvae.

	**pHLS9 [25 μM]**	**HepaRG [25 μM]**	**HepaRG [250 μM]**	**Zebrafish larvae**
**4F-MDMB-BINACA**				
Phase I	15	7	12	14
Phase II	2	2	3	4
**3,4-DMA-NBOMe**				
Phase I	6 (Caspar et al., 2018b)	6	10	9
Phase II	5 (Caspar et al., 2018b)	4	7	7
**Ephylone**				
Phase I	5 (Wagmann et al., 2019b)	2	4	5
Phase II	8 (Wagmann et al., 2019b)	4	6	4
**4F-PHP**				
Phase I	6 (Wagmann et al., 2019b)	5	6	8
Phase II	2 (Wagmann et al., 2019b)	0	1	3
**1P-LSD**				
Phase I	8 (Wagmann et al., 2019c)	14	15	22
Phase II	0 (Wagmann et al., 2019c)	1	2	3
**Total**				
Phase I	40	34	47	58
Phase II	17	11	19	21

The HRMS^2^ spectrum of 4F-MDMB-BINACA (PI at *m/z* 364.2031, C_19_H_27_O_3_N_3_F^+^) showed the subsequent fragmentation of the ester moiety due to an initial methanol loss (−32.0262 u, CH_4_O) forming the fragment ion (FI) at *m/z* 332.1769 (C_18_H_23_O_2_N_3_F^+^) followed by a loss of CO (−27.9949 u), resulting in the FI at *m/z* 304.1820 (C_17_H_23_ON_3_F^+^). The most abundant FI at *m/z* 219.0928 (C_12_H_12_ON_2_F^+^) corresponded to a cleavage of the amide moiety between the carbonyl carbon atom and the nitrogen atom. The FI at *m/z* 237.1034 (C_12_H_14_O_2_N_2_F^+^) represented a mass shift of +18.0106 u corresponding to the mass of water. Richter et al. postulated a rearrangement reaction for 5F-ADB potentially also explaining the current finding. 5F-ADB obtained a similar structure, but with a fluoro pentyl chain instead of the fluoro butyl chain of 4F-MDMB-BINACA. Ester cleavage followed by nucleophilic attack of the oxygen at the indazole nitrogen in position 2 may lead to cyclization (Richter et al., 2019b). Further cleavage of the amide bond and the ester bond may result in the FI at *m/*z 237.1034. The same shift was detected for the FIs at *m/z* 145.0396 (C_8_H_5_${\text{ON}}_{2}^{+}$) and 163.0502 (C_8_H_7_O_2_${\text{N}}_{2}^{+}$), formed after cleavage of the amide bond between the carbonyl carbon atom and the nitrogen atom and elimination of the fluoro butyl chain.

Due to the large number of metabolites tentatively identified in this study, only some 4F-MDMB-BINACA metabolites with fragmentation patterns that were representative for the other metabolites could be described in detail. After ester hydrolysis (MB8, PI at *m/z* 350.1874, C_18_H_25_O_3_N_3_F^+^), an initial loss of water (−18.0105 u, H_2_O) was observed resulting in the FI at *m/z* 332.1769 (C_18_H_23_O_2_N_3_F^+^), instead of a loss of methanol as described for the parent compound 4F-MDMB-BINACA, but also followed by a loss of CO. A similar fragmentation pattern was already described for other carboxylic acids (Niessen and Correa, 2016; Wagmann et al., 2019a). All other FIs were the same as those detected in the HRMS^2^ spectrum of 4F-MDMB-BINACA.

Two metabolites with the PI at *m/z* 366.1824 (MB13, MB14, C_18_H_25_O_4_N_3_F^+^) were identified as products of ester hydrolysis plus hydroxylation. Both spectra obtained an initial water loss resulting in the FI at *m/z* 348.1718 (C_18_H_23_O_3_N_3_F^+^) supporting the assumption of prior ester hydrolysis. Furthermore, the mass shift of +15.9949 u between the FI at *m/z* 320.1769 (C_17_H_23_O_2_N_3_F^+^) and 304.1820 (C_17_H_23_ON_3_F^+^) in the spectrum of the parent compound indicated an additional oxygen atom at the fluoro butyl chain, the indazole ring, or the *tert*-butyl group. In case of MB13, FIs at *m/z* 237.1034, 219.0928, 163.0502, and 145.0396 were detected, according to the HRMS^2^ spectrum of the parent compound, indicating unchanged fluoro butyl and indazole parts. Therefore, MB13 was hydroxylated at the *tert*-butyl group. In case of MB14, the FIs at *m/z* 253.0983, 235.0877, 179.0451, and 161.0346 represented the aforementioned FIs shifted by +15.9949 u (oxygen), indicating that the indazole part of MB14 was hydroxylated, but the exact position could not be determined based on the fragmentation pattern.

The PI at *m/z* 348.1718 of MB6 (C_18_H_23_O_3_N_3_F^+^) provided a mass shift of −2.0157 u regarding the PI of MB8 (ester hydrolysis). This was postulated to be an ester hydrolysis followed by dehydrogenation (Haschimi et al., 2019; Krotulski et al., 2019). Another possibility would be the formation of a lactone after hydroxylation of the *tert*-butyl part, with or without former ester hydrolysis as postulated for MDMB-FUBINACA (Kavanagh et al., 2017). The formation of the lactone was expected to be more likely due to the FI at *m/z* 320.1769 (C_17_H_23_O_2_N_3_F^+^), which was also detected for MB13 (ester hydrolysis + hydroxylation of the *tert-*butyl part) and MB16 (hydroxylation of the *tert-*butyl part), but not MB8 (ester hydrolysis), and the FI at *m/z* 113.0597 (C_6_H_9_${\text{O}}_{2}^{+}$), a 4,4-dimethyl-2-oxotetrahydrofuran-3-ylium ion, formed by cleavage of the bond between amide nitrogen and alpha carbon of the lactone part. In accordance with the observations of Kavanagh et al. (2017), MB6 was not ionizable in negative ionization mode, which contrasts with the characteristic behavior of carboxylic acids such as MB8 (ester hydrolysis).

Glucuronidation was identified by the increase of 176.0320 u (C_6_H_8_O_6_) in the parent mass. MB23 with the PI at *m/z* 542.2144 (C_24_H_33_O_10_N_3_F^+^) was identified as product of ester hydrolysis, hydroxylation of the indazole part, and glucuronidation. MB14 represented the corresponding phase I metabolite. MB14 and MB23 provided the same fragmentation pattern below *m/z* 366.1824 (PI of MB14), but differences in the abundance of several FIs. The FI at *m/z* 496.2089 (C_23_H_31_O_8_N_3_F^+^) in the HRMS^2^ spectrum of MB23 represented the FI at *m/z* 320.1769 (C_17_H_23_O_2_N_3_F^+^) conjugated with glucuronic acid. Therefore, the glucuronic acid moiety was most likely bound to the hydroxy group at the indazole part and not to the carboxylic acid.

According to the recommendation of Richter et al., two incubation time points were analyzed to detect initial metabolic steps as well as late phase metabolites (Richter et al., 2017b). As no reference standards of the metabolites were available, their quantification was not possible. Only their abundance could be compared to each other. However, it must be noticed that the highest concentration is not always reflected by the highest abundance, which is influenced by the ionizability of a compound. The most abundant metabolite in pHLS9 incubations after 1 and 6 h was MB8 (ester hydrolysis). After 1 h of incubation, it was followed by MB19 (hydroxylation of the indazole part isomer 2) and MB4 (*N*-dealkylation). After 6 h of incubation, MB6 (lactone formation) and MB15 (oxidative defluorination + oxidation to carboxylic acid) represented the second and third most abundant metabolites, respectively.

Incubations with pHLS9 following the same procedure were already performed for 3,4-DMA-NBOMe, ephylone, 4F-PHP, and 1P-LSD, and the results and their fragmentation patterns were described elsewhere (Caspar et al., 2018b; Wagmann et al., 2019b,c).

### *In vivo* Maximum-Tolerated Concentration Studies in Zebrafish Larvae

To exclude toxic effects of the investigated NPS on zebrafish larvae, which might prevent metabolic transformations, the MTC was evaluated. The survival rates of zebrafish larvae (4 dpf) after 24-h treatment with the NPS added to the medium at the concentrations of 0, 0.01, 0.1, 1, 10, 50, or 100 μM are given in Table 3. In case of 3,4-DMA-NBOMe, ephylone, 4F-PHP, and 1P-LSD, survival rates were 100% for all tested concentrations and no malformations were observed. Therefore, a concentration of 100 μM was considered to be non-toxic for zebrafish larva 4 dpf and chosen for metabolism studies. The MTC study with 4F-MDMB-BINACA showed survival rates of 73% and 53% at 50 and 100 μM, respectively. No malformations were observed. Further zebrafish studies were performed with only 25 μM 4F-MDMB-BINACA, and larvae were monitored in terms of survival and malformations, to ensure nontoxicity of the chosen concentration.

**Table 3:** Survival rates of zebrafish larvae (4 dpf) after 24-h treatment with various concentrations of 3,4-DMA-NBOMe, ephylone, 4F-MDMB-BINACA, 4F-PHP, or 1P-LSD, respectively.

**Concentration, μM**	**Survival rate, %**				
	**3,4-DMA-NBOMe**	**Ephylone**	**4F-MDMB-BINACA**	**4F-PHP**	**1P-LSD**
0	100	100	100	100	100
0.01	100	100	100	100	100
0.1	100	100	100	100	100
1	100	100	100	100	100
10	100	100	100	100	100
50	100	100	73	100	100
100	100	100	53	100	100

*Fifteen larvae were tested per concentration level and test compound, respectively*.

### Identification of Metabolites in Incubations With HepaRG Cells and Zebrafish Larvae and Comparison to Metabolites Identified in Incubations With pHLS9

Zebrafish were found to have similar metabolic enzymes to mammals and to be able to perform both phase I (such as oxidations, *N*-demethylations, *O*-demethylations, or *N*-dealkylations) and phase II metabolic reactions (for example, sulfations, glucuronidations, or methylations) (De Souza Anselmo et al., 2017; Matos et al., 2019). As whole organisms, zebrafish larvae may also overcome the limitations of *in vitro* model systems (Van Wijk et al., 2016). Concerning *in vitro* metabolism studies, human liver tissue-derived primary hepatocytes are considered as “gold standard,” but provide several disadvantages such as complex isolation procedures, high variability in the enzyme expression, and limited availability (Guillouzo et al., 2007; Godoy et al., 2013). Relevant alternative *in vitro* systems are sought after and especially HepaRG cells were found to be a suitable alternative to primary human hepatocytes (Lubberstedt et al., 2011; Godoy et al., 2013).

HepaRG cells represent immortalized human liver cells derived from a tumor. They were shown to remain capable of expressing most of the liver-specific functions, including the major CYPs involved in drug metabolism, but were derived from a CYP2D6 poor metabolizer patient (Guillouzo et al., 2007). Neither HepaRG cells nor zebrafish larvae experiments demand the supplementation of co-substrates of metabolic reactions representing their main advantage over pHLS9 incubations (Richter et al., 2017a). However, handling of cells as well as zebrafish larvae is time-consuming and requires special laboratory equipment and qualified personnel. Furthermore, zebrafish larvae experiments required a higher total amount of the NPS then HepaRG incubations. Incubations with pHLS9 could be performed with the lowest NPS amount, which might be beneficial in cases only small volumes of substances are available.

Based on the results of the MTC study, a 4F-MDMB-BINACA concentration of 25 μM was added to the zebrafish larvae medium and neither malformations nor dead larvae were observed after 24 h of incubation. Thus, 25 μM 4F-MDMB-BINACA was considered as non-toxic for zebrafish larvae (4 dpf). Focusing on the detection of metabolites, a previous study demonstrated the extraction of zebrafish larvae to be superior to the precipitation of the surrounding medium (Richter et al., 2019a). In case of HepaRG cell incubations, the precipitated cell culture medium was shown to be an appropriate matrix for identification of metabolites (Richter et al., 2017a, 2019a,c). HepaRG cell experiments were conducted using two different concentrations (25 and 250 μM, respectively) in accordance with a previous study (Richter et al., 2019c). All tentatively identified metabolites of 4F-MDMB-BINACA along with their absolute peak areas in HepaRG cells incubations or zebrafish larvae are listed in Table 1. The given areas were taken from one replicate of the HepaRG incubations (*n* = 3) or zebrafish larvae experiments (*n* = 3). The other replicates provided the same pattern of metabolite abundances. HRMS^2^ spectra of the parent compound and the metabolites are depicted in Figure 2 or Figure S1 in the ESM.

Nine metabolites (7 phase I, 2 phase II) were detected in the HepaRG cell culture medium using a 4F-MDMB-BINACA concentration of 25 μM and 15 metabolites (12 phase I, 3 phase II) were detected using 250 μM 4F-MDMB-BINACA (see Table 2). All metabolites identified after administration of 25 μM 4F-MDMB-BINACA to HepaRG cells could also be detected after administration of 250 μM as well as six additional metabolites. Most abundant metabolites were MB8 (ester hydrolysis) followed by MB7 (ester hydrolysis + oxidative defluorination) and MB22 (ester hydrolysis + glucuronidation). Eighteen metabolites (14 phase I, 4 phase II) were detected in zebrafish larvae (see Table 2). MB8 (ester hydrolysis), MB6 (lactone formation), and MB15 (oxidative defluorination + oxidation to carboxylic acid) provided the highest abundance. In conclusion, zebrafish larvae led to the identification of the highest number of 4F-MDMB-BINACA metabolites among the investigated models. In comparison to HepaRG cells, zebrafish larvae produced more phase I, but also II metabolites. Incubations with pHLS9 produced one phase I metabolite more but two phase II metabolites less than zebrafish larvae.

All tentatively identified metabolites of 3,4-DMA-NBOMe, ephylone, 4F-PHP, and 1P-LSD in HepaRG cell incubations and zebrafish larvae are listed in Tables S1–S4 (ESM), which also contain the metabolites detected in pHLS9 incubations described in literature. Detailed information on their MS fragmentation patterns may also be found in literature (Caspar et al., 2018b; Wagmann et al., 2019b,c). HRMS^2^ spectra of the parent compounds and the metabolites recorded during the current study are depicted in Figures S2–S5 (ESM). In accordance with the results for 4F-MDMB-BINACA, zebrafish larvae also produced the highest number of phase I and II metabolites of 4F-PHP and 1P-LSD. Concerning 3,4-DMA-NBOMe, most metabolites were formed by HepaRG cells with 250 μM substrate concentration, while pHLS9 incubations produced the highest number of ephylone metabolites.

Summarizing the results of all five investigated NPS (see Table 2), the highest number of metabolites could be detected in zebrafish larvae (79 metabolites in total, 58 phase I, 21 phase II). However, some metabolites were exclusively formed by zebrafish larvae (e.g., MB2, MB12, MN9, and MP3), possibly indicating species differences between humans and zebrafish. HepaRG cells after administration of 250 μM substrate were identified as the second most efficient metabolizing model (66 metabolites in total, 47 phase I, 19 phase II), followed by pHLS9 incubations (57 metabolites in total, 40 phase I, 17 phase II). In the current study, HepaRG cells produced more metabolites after application of 250 μM NPS in comparison to 25 μM. However, as the investigated NPS were not tested for toxicity in HepaRG cells prior to the experiments, incubation of two different concentrations is also recommended for future studies.

### Monooxygenases Activity Screening of 4F-MDMB-BINACA

The involvement of monooxygenases in the metabolic phase I transformations of 4F-MDMB-BINACA was elucidated using single-enzyme incubations. Single-enzyme incubations allowed the evaluation of the involvement of an individual isoform, which is important to assess the risk of interindividual differences based on polymorphisms or interactions with co-consumed drugs (of abuse) or food ingredients due to inhibition or induction (Meyer, 2016). Ten different CYP isoenzymes and FMO3 were used representing monooxygenases known to be involved in the human hepatic metabolism of xenobiotics (Wagmann et al., 2016). Incubations with pHLM, containing all these isozymes in their natural composition, were used as positive controls. Results are given in Table 4. The initial monooxygenases activity screenings of 3,4-DMA-NBOMe, ephylone, 4F-PHP, and 1P-LSD were described elsewhere (Caspar et al., 2018a; Wagmann et al., 2019b,c). CYP3A4 was described to be involved in the *N*-dealkylation and CYP2C19 and CYP2D6 in the *O*-demethylation and hydroxylation of 3,4-DMA-NBOMe (Caspar et al., 2018a; Wagmann et al., 2019b,c). Ephylone and 4F-PHP were both metabolized by CYP1A2, CYP2B6, CYP2C19, and CYP3A4 (Caspar et al., 2018a; Wagmann et al., 2019b,c), while 1P-LSD was *N*-dealkylated and hydroxylated exclusively by CYP3A4 (Caspar et al., 2018a; Wagmann et al., 2019b,c).

**Table 4:** Detection of the 4F-MDMB-BINACA phase I metabolites (metabolite IDs correspond to Table 1) in *in vitro* incubations containing 1 out of 11 monooxygenase isozymes, respectively, or pooled human liver microsomes (pHLM).

**Enzyme preparation**	**Metabolite ID and metabolic reaction**								
	**MB1 (Amide hydrolysis)**	**MB4 (*N*-Dealkylation)**	**MB6 (Lactone formation)**	**MB8 (Ester hydrolysis)**	**MB11 (Oxidative defluorination)**	**MB16 (Hydroxylation of the *tert*-butyl part)**	**MB17 (Hydroxylation of the butyl chain)**	**MB18 (Hydroxylation of the indazole part isomer 1)**	**MB19 (Hydroxylation of the indazole part isomer 2)**
CYP1A2	–	+	–	–	–	–	–	–	+
CYP2A6	–	–	–	–	–	–	–	–	–
CYP2B6	–	–	–	–	–	–	–	–	–
CYP2C8	–	–	–	–	+	–	–	–	–
CYP2C9	–	–	–	–	–	–	–	–	–
CYP2C19	–	+	–	–	–	–	+	+	+
CYP2D6	–	–	–	–	–	–	–	–	–
CYP2E1	–	–	–	–	–	–	–	–	–
CYP3A4	–	+	+	–	–	–	+	+	–
CYP3A5	–	+	+	–	+	+	+	+	+
FMO3	–	–	–	–	–	–	–	–	–
pHLM	+	+	+	+	+	+	+	+	+

*Incubations were performed in duplicate. CYP, cytochrome P450; FMO, flavin-containing monooxygenase; +, detected; −, not detected*.

CYP1A2, CYP2C8, CYP2C19, CYP3A4, and CYP3A5 were shown to be involved in the metabolic phase I reactions of 4F-MDMB-BINACA. CYP3A5 was involved in all observed metabolic steps with the exception of the amide hydrolysis (MB1) and the ester hydrolysis (MB8). Both hydrolysis steps were found to be not catalyzed by the investigated monooxygenases but, interestingly, only detected in pHLM incubations. The elimination of the fluoro butyl chain (MB4) was catalyzed by CYP1A2, CYP2C19, CYP3A4, and CYP3A5. The lactone formation (MB6) was catalyzed by CYP3A4 and CYP3A5, demonstrating that previous ester hydrolysis was not essential for formation of MB6. The oxidative defluorination (MB11) was detected in incubations with CYP2C8 and CYP3A5. Hydroxylated metabolites (MB16–MB19) were formed by CYP1A2, CYP2C19, CYP3A4, and CYP3A5. Due to the high number of CYP isozymes found to be involved in the phase I metabolism, it is unlikely that polymorphisms or inhibition of a single isozyme will have a high impact on the biotransformation of 4F-MDMB-BINACA *in vivo*.

### Esterase Activity Screening of 4F-MDMB-BINACA

In order to investigate the involvement of esterases in the metabolism of 4F-MDMB-BINACA, an esterase activity screening was performed using three recombinant human carboxylesterases, pHLM, pHLS9, and pooled human blood plasma. The results are listed in Table 5. Carboxylesterases are membrane-bound phase I drug-metabolizing enzymes that can hydrolyze a variety of xenobiotics (Wang et al., 2011). Ester hydrolysis of 4F-MDMB-BINACA (MB8) was shown to be catalyzed by hCES1b and hCES1c. This was consistent with a previous study by Meyer et al., describing that drugs of abuse with small alcohol parts were mainly hydrolyzed by hCES1 isoforms (Meyer et al., 2015). Amide hydrolysis (MB1) was shown to be catalyzed by hCES1c. MB1 and MB8 were also detected in incubations with pHLM and pHLS9, but not in incubations with pooled human blood plasma. The human liver preparations of pHLM and pHLS9 are known to contain carboxylesterases, mainly hCES1 isoforms, but also hCES2 in lower levels (Wang et al., 2011). Pooled human blood plasma contains no carboxylesterases, but the four non-specific esterases such as butyrylcholinesterase, paraoxonase, acetylcholinesterase, and albumin esterase (Li et al., 2005). MB1 was not identified in the prior pHLS9 incubations for identification of phase I and II metabolites of 4F-MDMB-BINACA. This was most probably caused by low formation levels of MB1 in these incubations due to the predominance of other metabolic pathways. Genetic polymorphisms of hCES1 have been shown to affect the metabolism of several drugs (Di, 2019). Therefore, interindividual differences in the hydrolysis of 4F-MDMB-BINACA cannot be excluded.

**Table 5:** Detection of the 4F-MDMB-BINACA metabolites after amide hydrolysis (MB1) or ester hydrolysis (MB8, metabolite IDs correspond to Table 1) in *in vitro* incubations containing one out of three human carboxylesterase isozymes (hCES), respectively, pooled human liver microsomes (pHLM), pooled human liver S9 fraction (pHLS9), or pooled human blood plasma (pHBP).

**Enzyme preparation**	**Metabolite ID**	
	**MB1**	**MB8**
hCES1b	–	+
hCES1c	+	+
hCES2	–	–
pHLM	+	+
pHLS9	+	+
pHBP	–	–
		

*Incubations were performed in duplicate; +, detected; –, not detected*.

### Comparison to Metabolites Identified in Human Biosamples

Zebrafish larvae were identified as a model system producing the highest number of metabolites of the five investigated NPS. However, a high number of metabolites does not necessarily mean that a model system is the most suitable one for developing analytical procedures for human biosamples. The metabolites identified during a metabolism study must be implemented in screening procedures for human biosamples aiming to detect the intake of NPS for example in a clinical or forensic toxicological context. The consideration of metabolites as targets is crucial particularly in urine screening (Wagmann and Maurer, 2018), but the detection of NPS can only be successful if the implemented metabolites were the same as the ones present in human biosamples. The implementation should not be motivated by the abundance of the metabolite. Each model system has limitations and a minor metabolite detected in a model system may be a major metabolite in humans. Metabolites tentatively identified in pHLS9, HepaRG cells, and zebrafish larvae incubations were compared to those detected in human biosamples to determine the measure of concordance.

Human blood and urine samples of a male individual after suspected intake of drugs of abuse were submitted to the authors' laboratory for regular toxicological analysis. The patient was initially found unconscious and taken to the hospital. A comprehensive toxicological screening in plasma and urine was performed including numerous therapeutic drugs, drugs of abuse, NPS, and their metabolites. The toxicological screening detected not only 4F-MDMB-BINACA but also amitriptyline and quetiapine at therapeutic levels in the patient's biosamples. No targeted analysis for detection of γ-hydroxybutyrate (GHB), which would also be able to cause deep sedation, was performed due to the patient's symptoms and the case history.

**Figure 3:**
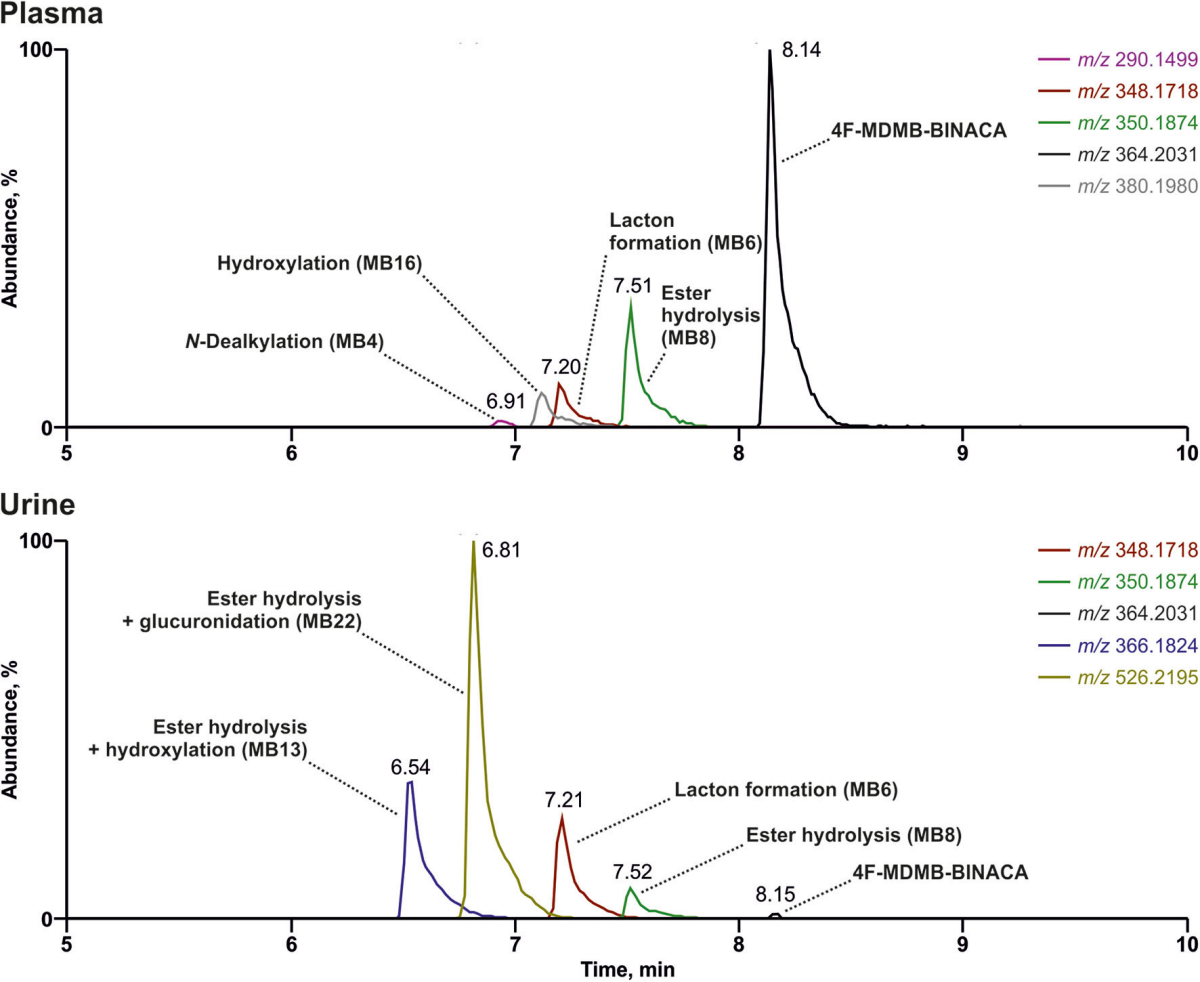
Reconstructed chromatograms of the mass-to-charge ratios of 4F-MDMB-BINACA and its metabolites in high-resolution full scan derived from an authentic human plasma and urine sample after unknown dose and time point of 4F-MDMB-BINACA intake. Colored figure may be viewed online.

The reconstructed chromatograms of the *m/z* of 4F-MDMB-BINACA and its metabolites derived from the HR full scan of the plasma and urine sample analysis are given in Figure 3. The most abundant signals in descending order in the plasma sample were assigned to the parent compound 4F-MDMB-BINACA, MB8 (ester hydrolysis), MB6 (lactone formation), and MB16 (hydroxylation of the *tert*-butyl part). Time point and dosage of the 4F-MDMB-BINACA intake were unknown, but the presence of the parent compound and the patient's health status suggested a recent intake. The most abundant signals in the urine sample were assigned to MB22 (ester hydrolysis + glucuronidation), MB13 (ester hydrolysis + hydroxylation of the *tert*-butyl part), MB6 (lactone formation), and MB8 (ester hydrolysis). However, the parent compound was barely detectable in urine. Therefore, MB6, MB8, and MB22 are particularly recommended as targets for comprehensive screening procedures, if the sample preparation of urine does not contain conjugate cleavage. Otherwise, MB22 does not have to be included. The approaches used by Haschimi et al. and Krotulski et al. included conjugate cleavage in urine and both only described phase I metabolites of 4F-MDMB-BINACA (Haschimi et al., 2019; Krotulski et al., 2019). Haschimi et al. identified 13 metabolites in 17 human urine samples and recommended MB6 and MB8 as reliable urinary markers (Haschimi et al., 2019). Krotulski et al. identified nine 4F-MDMB-BINACA metabolites in four blood and four urine samples and only recommended to include MB8 as well as MB11 in toxicological screening procedures (Krotulski et al., 2019).

Table 6 summarizes the concordance of the four most abundant signals in the current patient's plasma or urine with the 4F-MDMB-BINACA metabolites identified in pHLS9, HepaRG cells, and zebrafish larvae experiments. Concerning plasma, all four compounds reflecting the four most abundant signals were detectable in pHLS9 incubations and zebrafish larvae (100%), while only two (50%) or three compounds (75%) were detectable in HepaRG cell experiments depending on the used NPS concentration. HepaRG cells with 250 μM 4F-MDMB-BINACA formed all four most abundant metabolites in urine (100%), while pHLS9 and zebrafish larvae formed three metabolites (75%) and HepaRG with 25 μM 4F-MDMB-BINACA formed only two of them (50%). These findings were in accordance with the previous study focusing on another synthetic cannabinoid. Richter et al. reported HepaRG cells to form the highest number of the most abundant human urinary metabolites of 7′N-5F-ADB, followed by zebrafish larvae and pHLS9 incubations (Richter et al., 2019a).

**Table 6:** Relative number of matches (%) with the four most abundant signals detected in human plasma and urine samples after intake of 4F-MDMB-BINACA, ephylone, or 4F-PHP compared to incubations with pooled human liver S9 fraction (pHLS9), HepaRG cells, and zebrafish larvae.

	**pHLS9 [25 μM]**	**HepaRG [25 μM]**	**HepaRG [250 μM]**	**Zebrafish larvae**
**4F-MDMB-BINACA**				
Plasma	100	50	75	100
Urine	75	50	100	75
**Ephylone (Wagmann et al.**, **2019b****)**				
Plasma	100	75	100	100
Urine	100	75	100	100
**4F-PHP (Wagmann et al.**, **2019b****)**				
Plasma	75	50	50	100
Urine	75	25	50	75
**Total**	88	54	79	92

Data concerning detectability of ephylone and 4F-PHP in human biosamples were also available and taken from a recent publication (Wagmann et al., 2019b). Ephylone, ME1 (*N*-deethylation), ME2 (demethylenation), ME3/4 (demethylenation + methylation), as well as the corresponding glucuronic acid conjugates of ME2 (ME11/12) and ME3/4 (ME13/14) represented the highest ephylone-related signals in human plasma and/or urine. In case of 4F-PHP, the parent compound, MP2 (reduction), MP3 and MP4 (hydroxylation + oxidation to ketone or lactam), and MP10 (reduction + glucuronidation) led to the signals with the highest abundance (Wagmann et al., 2019b). As given in Table 6, pHLS9, HepaRG cells with 250 μM NPS, and zebrafish larvae experiments provided perfect agreement with the four most abundant ephylone-related signals in plasma and urine (100%). Zebrafish larvae experiments were also perfectly suited for detection of the four most abundant 4F-PHP-related signals in human plasma (100%). The accordance with human urine was the same for pHLS9 and zebrafish larvae experiments, but both models only led to the detection of three out of the four most abundant signals (75%). Overall, zebrafish larvae experiments provided the most comprehensive concordance of detected NPS-related compounds with those present in the human biosamples plasma and urine (92%). Incubations with pHLS9 came as second best (88%) followed by HepaRG cell incubations with 250 μM NPS (79%). These findings underline the suitability of zebrafish larvae for toxicokinetic studies of NPS. As zebrafish larvae represent intact organisms, their possible application is beyond metabolism studies, e.g., investigations on distribution or excretion patterns of NPS. However, the most easily manageable model investigated in this study, the pHLS9 incubations, also provided a good overlap with metabolites detected in human biosamples and can also be considered as an appropriate *in vitro* metabolism model for developing toxicological screening procedures.

Unfortunately, only very limited data concerning detectability of 3,4-DMA-NBOMe and 1P-LSD in human biosamples were available. Nevertheless, Caspar et al. recommended 3,4-DMA-NBOMe after *O*-demethylation (MN5 or MN6) or *O,O*-didemethylation (MN2) of the amphetamine part as well as their glucuronides (MN20 or MN21 and MN17) as urine screening targets for 3,4-DMA-NBOMe after oral application to rats. HepaRG cells and zebrafish larvae produce MN5, MN6, MN20, and MN21, while Caspar et al. only detected one isomer (MN5 or MN6 and MN20 or MN21), but also MN2 and MN17 in pHLS9 incubations (Caspar et al., 2018b).

It is likely that 1P-LSD is hydrolyzed to LSD *in vivo* (Brandt et al., 2016; Grumann et al., 2019; Halberstadt et al., 2019; Wagmann et al., 2019c). Grumann et al. reported that 1P-LSD could not be detected in serum and urine samples of an intoxicated patient after assumed consumption of a blotter containing 1P-LSD. However, they were able to find LSD in both matrices, but no information on the presence of 1P-LSD-specific metabolites in the biosamples was given (Grumann et al., 2019). Halberstadt et al. were able to detect 1P-LSD, LSD, and several metabolites in rat plasma after subcutaneous administration to rats. Some metabolites were found to be 1P-LSD specific as they still contained the 1-propionyl moiety (Halberstadt et al., 2019).

Finally, some limitations of the present study must be considered. First, only biosamples of one individual after intake of 4F-MDMB-BINACA or ephylone and 4F-PHP were used for comparison and interindividual metabolism differences cannot be excluded. The same is true for species differences between humans and zebrafish concerning the metabolism of 3,4-DMA-NBOMe and 1P-LSD, as human biosamples were not available. In addition, different NPS concentrations were used for the different metabolism models based on experience and MTC study results.

## Conclusions

The three metabolism models, by name pHLS9 incubations, HepaRG cells, and zebrafish larvae, formed numerous metabolites of the five NPS. Zebrafish larvae were found to produce the highest number of metabolites. The 4F-MDMB-BINACA, ephylone, and 4F-PHP metabolites formed by zebrafish larvae also provided the highest concordance with the four most abundant signals in human biosamples. Nevertheless, all metabolism models produced metabolites similar to those detected in authentic biosamples. Furthermore, several CYP isozymes as well as hCES1b and hCES1c were shown to be involved in the phase I metabolic reactions of 4F-MDMB-BINACA. Interindividual differences, presumably mainly based on hCES1 polymorphisms, cannot be excluded. The current study underlines the potential of zebrafish larvae as a tool for elucidating the toxicokinetics of NPS in the future.

## Data Availability Statement

Datasets generated for this study are included in the article/Supplementary Material or available upon reasonable request.

## Ethics Statement

Ethical review and approval was not required for the study on human participants in accordance with the local legislation and institutional requirements. Written informed consent for participation was not required for this study in accordance with the national legislation and the institutional requirements.

## Author Contributions

LW and MM established the study design and planned the experiments. FF and LW were responsible for the experimental execution. JH, YP, and RM were responsible for Zebrafish larvae experiments. VF and LW were responsible for HepaRG cell experiments. SF and FW provided reference standard of 4F-MDMB-BINACA. LW and FF wrote the first draft of the manuscript. All authors contributed to manuscript revision, read, and approved the submitted version.

## Conflict of Interest

The authors declare that the research was conducted in the absence of any commercial or financial relationships that could be construed as a potential conflict of interest.
