# Supplementary material for: How to Study the Metabolism of New Psychoactive Substances for the Purpose of Toxicological Screenings—A Follow-Up Study Comparing Pooled Human Liver S9, HepaRG Cells, and Zebrafish Larvae
Source: Front Chem. 2020 Jul 17;8:539. doi: 10.3389/fchem.2020.00539 (PMC7380166; doi:10.3389/fchem.2020.00539)
Supplement: Supplementary file 1 [file Data_Sheet_1.PDF]

How to study the metabolism of new psychoactive substances for the purpose of toxicological screenings? − A follow−up study comparing pooled human liver S9, HepaRG cells, and zebrafish larvae

Lea Wagmann, Fabian Frankenfeld, Yu Mi Park, Jennifer Herrmann, Svenja Fischmann, Folker Westphal, Rolf Müller, Veit Flockerzi, and Markus R. Meyer

Electronic Supplementary Material

Table S1 Detection of 3,4−DMA−NBOMe and its phase I and II metabolites in incubations with pooled human liver S9 fraction (pHLS9, *n* = 2, according to literature), HepaRG cells (*n* = 3), and zebrafish larvae (*n* = 3) along with the unique metabolite ID, observed metabolic reaction, and calculated exact mass. The absolute peak areas in HepaRG or zebrafish larvae experiments were taken from one replicate and the five largest areas per model are given in bold. MN, 3,4−DMA−NBOMe metabolite; + or area, detected; n.d., not detected

Metabolite ID	Metabolic reaction	Calculated exact masses, *m/z*	pHLS9 [25 µM] (Caspar et al., 2018)	HepaRG [25 µM]	HepaRG [250 µM]	Zebrafish larvae [100 µM]
3,4−DMA−NBOMe	− (parent compound)	316.1907	+	**1.48E+09**	**1.03E+10**	**6.22E+09**
MN1	*N*−Dealkylation	196.1332	+	**4.04E+07**	**9.84E+07**	**3.29E+07**
MN2	*O,O*−Didemethylation (amphetamine)	288.1594	+	n.d.	n.d.	n.d.
MN3	*O,O*−Didemethylation (amphetamine + NBOMe) isomer 1	288.1594	n.d.	n.d.	3.94E+05	3.21E+05
MN4	*O,O*−Didemethylation (amphetamine + NBOMe) isomer 2	288.1594	n.d.	n.d.	6.81E+05	n.d.
MN5	*O*−Demethylation (amphetamine) isomer 1	302.1751	+ (or MN6)	**4.44E+06**	1.80E+07	2.16E+06
MN6	*O*−Demethylation (amphetamine) isomer 2	302.1751	+ (or MN5)	2.05E+06	1.67E+07	**2.54E+07**
MN7	*O*−Demethylation (NBOMe)	302.1751	+	**1.19E+07**	**1.17E+08**	**1.19E+08**
MN8	*O*−Demethylation (NBOMe) + hydroxylation (NBOMe)	318.1700	n.d.	n.d.	6.72E+05	3.05E+05
MN9	*O*−Demethylation (NBOMe) + *N*−oxygenation	318.1700	n.d.	n.d.	n.d.	3.57E+05
MN10	Hydroxylation (NBOMe)	332.1856	+	1.01E+06	4.58E+06	1.59E+07
MN11	Hydroxylation (amphetamine)	332.1856	+	3.73E+06	2.11E+07	n.d.
MN12	*N*−Oxygenation	332.1856	n.d.	n.d.	4.60E+05	8.81E+06
MN13	*O*−Demethylation (amphetamine) + sulfation isomer 1	382.1319	+ (or MN14)	n.d.	**3.02E+07**	2.55E+06
MN14	*O*−Demethylation (amphetamine) + sulfation isomer 2	382.1319	+ (or MN13)	n.d.	n.d.	3.03E+06
MN15	*O*−Demethylation (NBOMe) + hydroxylation (NBOMe) + sulfation	398.1268	n.d.	n.d.	n.d.	4.12E+05
MN16	Hydroxylation (NBOMe) + sulfation	412.1424	n.d.	2.64E+05	1.68E+06	4.01E+06
MN17	*O,O*−Didemethylation (amphetamine) + glucuronidation	464.1915	+	n.d.	n.d.	n.d.
MN18	*O,O*−Didemethylation (amphetamine + NBOMe) + glucuronidation isomer 1	464.1915	n.d.	n.d.	1.20E+05	n.d.
MN19	*O,O*−Didemethylation (amphetamine + NBOMe) + glucuronidation isomer 2	464.1915	n.d.	n.d.	6.39E+05	n.d.
MN20	*O*−Demethylation (amphetamine) + glucuronidation isomer 1	478.2072	+ (or MN21)	**7.53E+06**	**2.30E+07**	**3.49E+07**

MN21	*O*−Demethylation (amphetamine) + glucuronidation isomer 2	478.2072	+ (or MN20)	1.33E+06	6.45E+06	7.17E+06
MN22	*O*−Demethylation (NBOMe) + glucuronidation	478.2072	+	n.d.	n.d.	n.d.
MN23	Hydroxylation (amphetamine) + glucuronidation	508.2177	+	n.d.	n.d.	n.d.
MN24	Hydroxylation (NBOMe) + glucuronidation	508.2177	n.d.	1.86E+06	4.86E+06	1.80E+06

Table S2 Detection of ephylone its phase I and II metabolites in incubations with pooled human liver S9 fraction (pHLS9, *n* = 2, according to literature), HepaRG cells (*n* = 3), and zebrafish larvae (*n* = 3) along with the unique metabolite ID, observed metabolic reaction, and calculated exact mass. The absolute peak areas in HepaRG or zebrafish larvae experiments were taken from one replicate and the five largest areas per model are given in bold. ME, ephylone metabolite; + or area, detected; n.d., not detected

Metabolite ID	Metabolic reaction	Calculated exact masses, *m/z*	pHLS9 [25 µM] (Wagmann et al., 2019a)	HepaRG [25 µM]	HepaRG [250 µM]	Zebrafish larvae [100 µM]
Ephylone	− (parent compound)	250.1438	+	**6.38E+08**	**3.94E+09**	**3.57E+09**
ME1	*N*−Deethylation	222.1125	+	**4.14E+06**	**2.21E+07**	4.12E+06
ME2	Demethylenation	238.1438	+	1.72E+05	6.37E+05	4.44E+06
ME3	Demethylenation + methylation isomer 1	252.1594	+	**1.96E+06**	**7.36E+06**	n.d.
ME4	Demethylenation + methylation isomer 2	252.1594	+	**2.48E+06**	**1.11E+07**	**1.17E+07**
ME5	Reduction	252.1594	+	n.d.	n.d.	**7.64E+06**
ME6	Hydroxylation	266.1387	+	n.d.	1.37E+06	**1.48E+07**
ME7	*N*−Oxygenation	266.1387	+	n.d.	1.75E+06	1.10E+06
ME8	Demethylenation + sulfation isomer 1	318.1006	+	n.d.	n.d.	n.d.
ME9	Demethylenation + sulfation isomer 2	318.1006	+	n.d.	n.d.	n.d.
ME10	Demethylenation + methylation + sulfation	332.1162	+	4.33E+05	2.23E+06	n.d.
ME11	Demethylenation + glucuronidation isomer 1	414.1759	n.d.	n.d.	3.00E+05	6.34E+05
ME12	Demethylenation + glucuronidation isomer 2	414.1759	+	n.d.	2.98E+05	n.d.
ME13	Demethylenation + methylation + glucuronidation isomer 1	428.1915	+	**2.86E+06**	**1.23E+07**	**1.21E+07**
ME14	Demethylenation + methylation + glucuronidation isomer 2	428.1915	+	n.d.	n.d.	1.88E+06

Table S3 Detection of 4F−PHP and its phase I and II metabolites in incubations with pooled human liver fraction (pHLS9, *n* = 2, according to literature), HepaRG cells (*n* = 3), and zebrafish larvae (*n* = 3) along with the unique metabolite ID, observed metabolic reaction, and calculated exact mass. The absolute peak areas in HepaRG or zebrafish larvae experiments were taken from one replicate and the five largest areas per model are given in bold. MP, 4F−PHP metabolite; + or area, detected; n.d., not detected

Metabolite ID	Metabolic reaction	Calculated exact masses, *m/z*	pHLS9 [25 µM] (Wagmann et al., 2019a)	HepaRG [25 µM]	HepaRG [250 µM]	Zebrafish larvae [100 µM]
4F−PHP	− (parent compound)	264.1758	+	**6.65E+08**	**5.45E+09**	**4.36E+09**
MP1	*N,N*−Dealkylation	210.1289	+	1.48E+05	1.82E+06	4.83E+05
MP2	Reduction	266.1915	+	**1.18E+07**	**6.41E+07**	**2.98E+07**
MP3	Hydroxylation + oxidation to ketone	278.1551	n.d.	n.d.	n.d.	7.58E+04
MP4	Hydroxylation + oxidation to lactam	278.1551	+	n.d.	n.d.	8.64E+05
MP5	Hydroxylation isomer 1	280.1707	+	**1.14E+06**	**1.07E+07**	**7.71E+06**
MP6	Hydroxylation isomer 2	280.1707	+	n.d.	n.d.	n.d.
MP7	Hydroxylation isomer 3	280.1707	+	**7.75E+06**	**4.22E+07**	1.41E+06
MP8	*N*−Oxygenation	280.1707	n.d.	n.d.	1.47E+05	**2.50E+06**
MP9	Reduction + hydroxylation	282.1864	n.d.	**8.15E+05**	**6.48E+06**	**3.78E+07**
MP10	Reduction + glucuronidation	442.2236	+	n.d.	2.91E+04	n.d.
MP11	Hydroxylation + glucuronidation	456.2028	+	n.d.	n.d.	1.15E+06
MP12	Reduction + hydroxylation + glucuronidation isomer 1	458.2185	n.d.	n.d.	n.d.	2.71E+05
MP13	Reduction + hydroxylation + glucuronidation isomer 2	458.2185	n.d.	n.d.	n.d.	4.14E+05

Table S4 Detection of 1P−LSD and its phase I and II metabolites in incubations with pooled human liver S9 fraction (pHLS9, *n* = 2, according to literature), HepaRG cells (*n* = 3), and zebrafish larvae (*n* = 3) along with the unique metabolite ID, observed metabolic reaction, and calculated exact mass. The absolute peak areas in HepaRG or zebrafish larvae experiments were taken from one replicate and the five largest areas per model are given in bold. ML, 1P−LSD metabolite; + or area, detected; n.d., not detected

Metabolite ID	Metabolic reaction	Calculated exact masses, *m/z*	pHLS9 [25 µM] (Wagmann et al., 2019b)	HepaRG [25 µM]	HepaRG [250 µM]	Zebrafish larvae [100 µM]
1P−LSD	− (parent compound)	380.2333	+	**3.60E+07**	5.41E+06	**2.92E+09**
ML1	Depropionylation + *N,N*−bisdeethylation	268.1444	n.d.	n.d.	1.93E+06	n.d.
ML2	Depropionylation + diethylamide hydrolysis	269.1285	n.d.	5.16E+06	**1.39E+07**	3.63E+05
ML3	Depropionylation + *N*−deethylation + *N*−demethylation	282.1601	n.d.	3.89E+05	2.20E+06	3.30E+05
ML4	Depropionylation + *N*−deethylation	296.1757	+	**3.19E+07**	**2.58E+08**	**6.17E+07**
ML5	Depropionylation + *N*−demethylation	310.1914	+	**1.75E+07**	**3.37E+07**	1.07E+07
ML6	Depropionylation + *N*−deethylation + hydroxylation	312.1707	n.d.	4.44E+05	2.19E+06	1.12E+06
ML7	Depropionylation (= LSD)	324.2070	+	**8.59E+08**	**5.95E+09**	**1.25E+09**
ML8	Diethylamide hydrolysis	325.1547	n.d.	n.d.	n.d.	1.07E+06
ML9	Depropionylation + *N*−demethylation + hydroxylation isomer 1	326.1863	n.d.	n.d.	n.d.	5.77E+04
ML10	Depropionylation + *N*−demethylation + hydroxylation isomer 2	326.1863	n.d.	n.d.	n.d.	6.10E+04
ML11	Depropionylation + *N*−deethylation + dihydroxylation	328.1656	n.d.	1.20E+05	1.47E+05	n.d.
ML12	*N*−Deethylation + *N*−demethylation	338.1863	n.d.	n.d.	n.d.	4.11E+05
ML13	Depropionylation + hydroxylation isomer 1	340.2020	+	1.01E+06	2.40E+06	7.94E+05
ML14	Depropionylation + hydroxylation isomer 2	340.2020	n.d.	4.01E+05	4.63E+06	3.90E+06
ML15	Depropionylation + hydroxylation isomer 3	340.2020	+	**1.85E+07**	**3.70E+07**	7.80E+06
ML16	Depropionylation + hydroxylation isomer 4	340.2020	n.d.	4.05E+06	7.14E+06	2.70E+06
ML17	*N*−Deethylation	352.2020	+	n.d.	n.d.	**1.48E+08**
ML18	Depropionylation + dihydroxylation isomer 1 (= 2−oxo−3−hydroxy LSD)	356.1969	+	7.00E+06	3.67E+06	6.93E+06
ML19	Depropionylation + dihydroxylation isomer 2	356.1969	n.d.	3.74E+06	1.33E+07	n.d.
ML20	*N*−Demethylation	366.2176	+	3.19E+04	3.74E+06	**3.77E+07**
ML21	*N*−Deethylation + hydroxylation	368.1969	n.d.	n.d.	n.d.	3.16E+06
ML22	*N*−Demethylation + hydroxylation	382.2125	n.d.	n.d.	n.d.	2.35E+05
ML23	Hydroxylation isomer 1	396.2282	n.d.	n.d.	n.d.	3.31E+07
ML24	Hydroxylation isomer 2	396.2282	n.d.	n.d.	n.d.	3.37E+07
ML25	Hydroxylation + *N*−oxygenation	412.2231	n.d.	n.d.	n.d.	2.04E+06
ML26	Depropionylation + hydroxylation + sulfation	420.1588	n.d.	n.d.	n.d.	3.62E+05
ML27	Depropionylation + hydroxylation + glucuronidation isomer 1	516.2340	n.d.	1.51E+05	1.98E+05	3.39E+06
ML28	Depropionylation + hydroxylation + glucuronidation isomer 2	516.2340	n.d.	n.d.	1.07E+05	n.d.
ML29	Hydroxylation + glucuronidation	572.2603	n.d.	n.d.	n.d.	1.12E+05

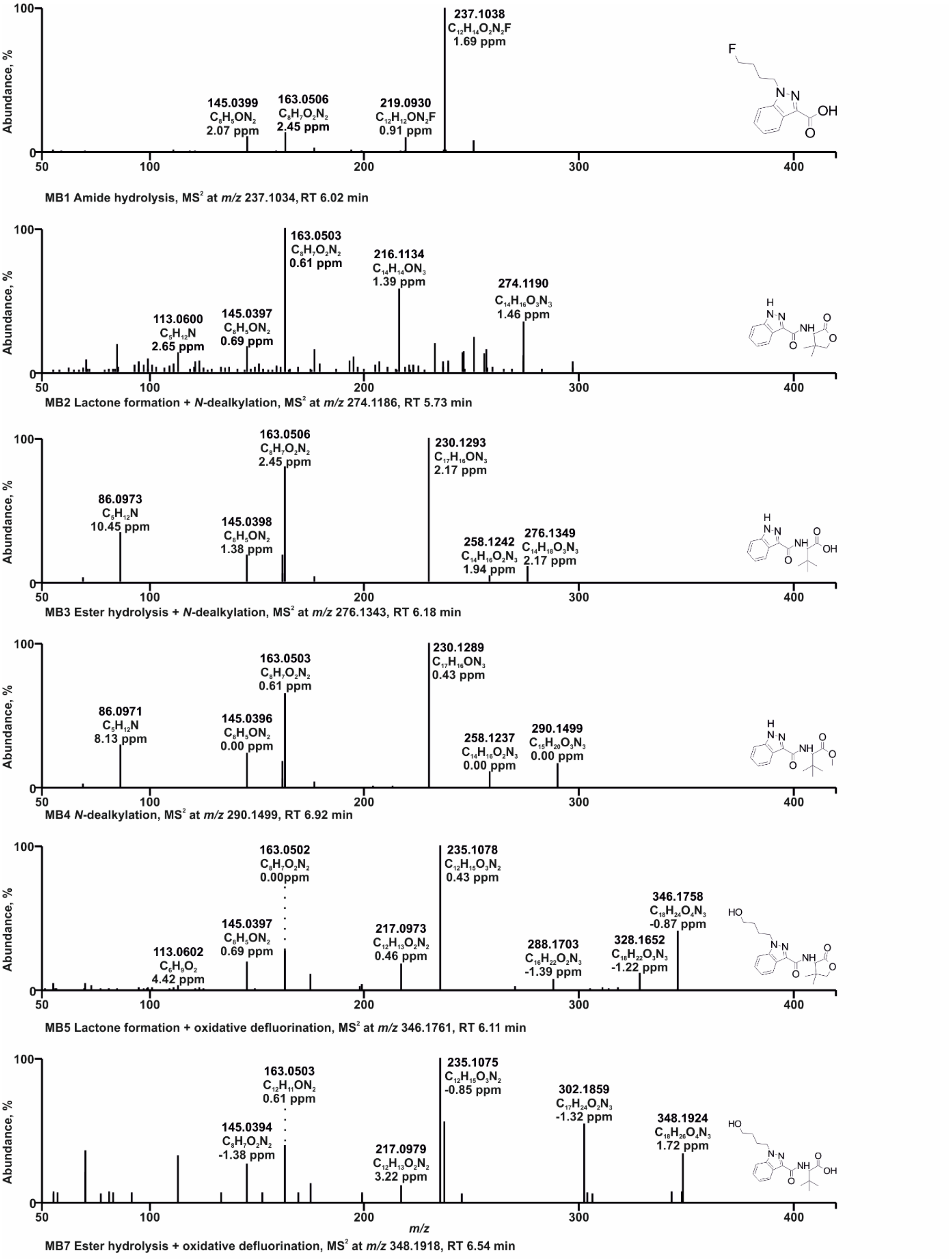

Figure S1 HRMS^2 spectra of 4F−MDMB−BINACA metabolites detected in incubations with pooled human liver S9 fraction, HepaRG cells, and/or zebrafish larvae. MB, 4F−MDMB−BINACA metabolite; RT, retention time

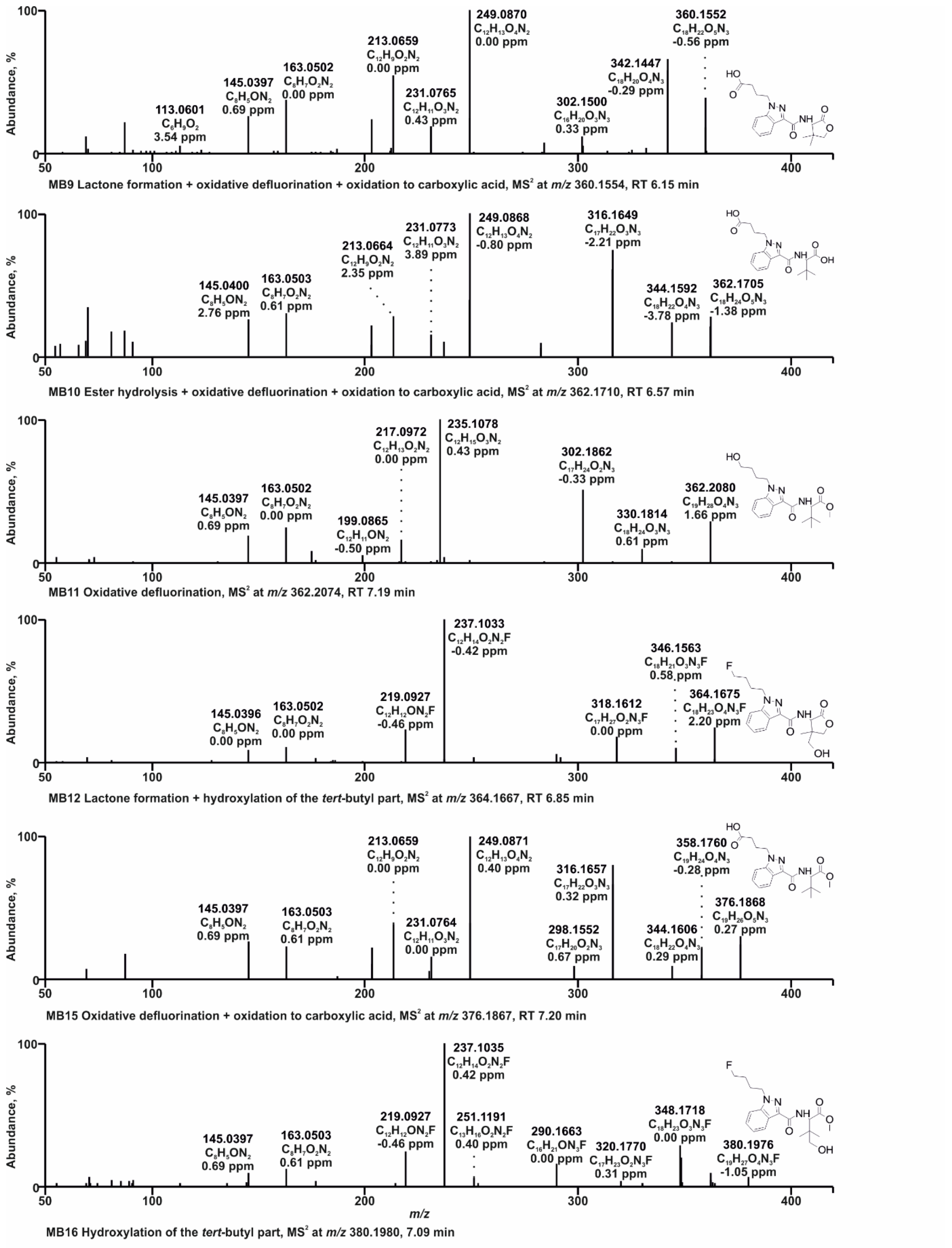

Figure S1 (continued)

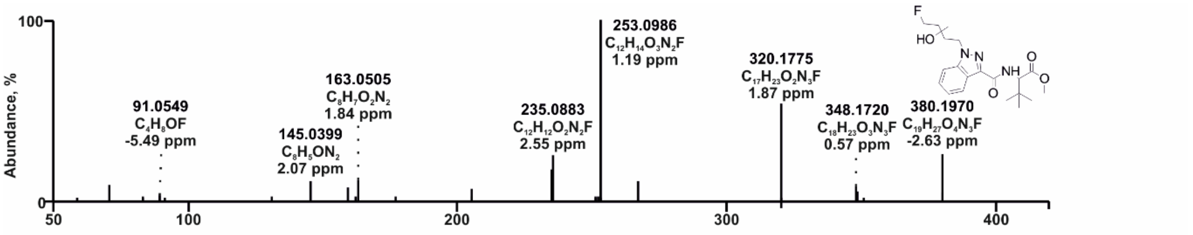

MB17 Hydroxylation of the butyl chain, MS^2 at *m/z* 380.1980, 7.46 min

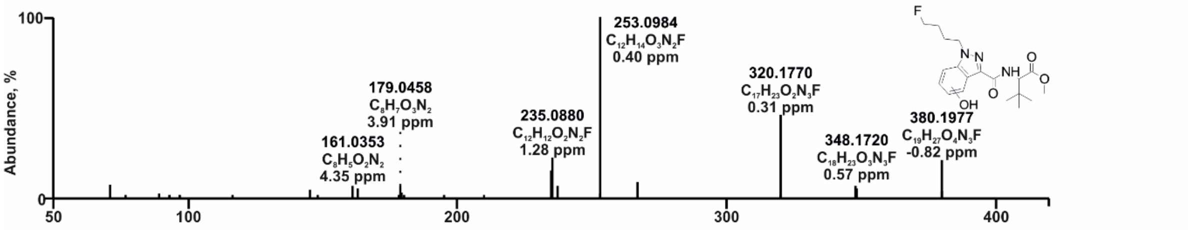

MB18 Hydroxylation of the indazole part isomer 1, MS^2 at *m/z* 380.1980, RT 7.55 min

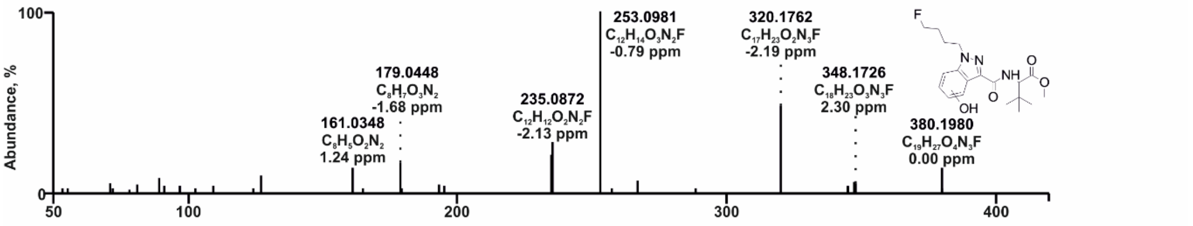

MB19 Hydroxylation of the indazole part isomer 2, MS^2 at *m/z* 380.1980, RT 7.67 min

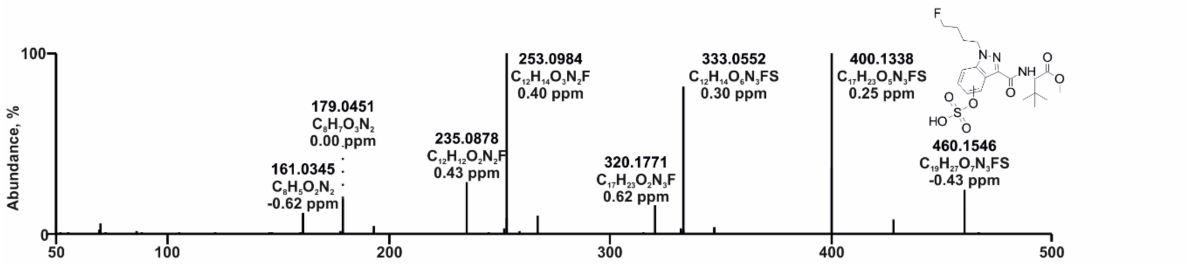

MB20 Hydroxylation of the indazole part + sulfation, MS^2 at *m/z* 460.1548, RT 6.88 min

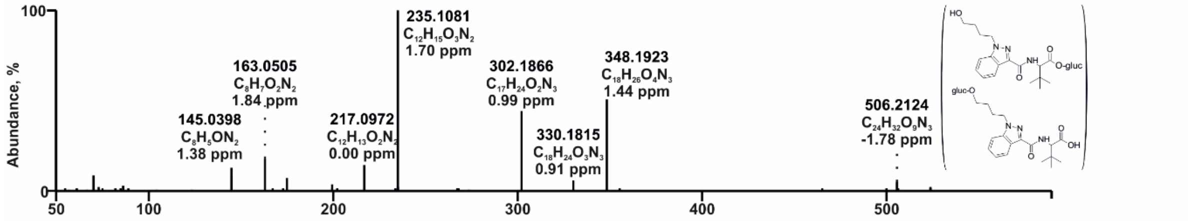

MB21 Ester hydrolysis + oxidative defluorination + glucuronidation, MS^2 at *m/z* 524.2239, RT 5.94 min

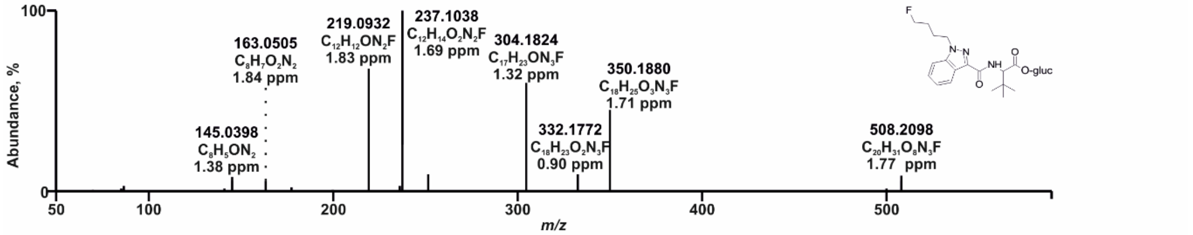

MB22 Ester hydrolysis + glucuronidation, MS^2 at *m/z* 526.2196, RT 6.81 min

Figure S1 (continued)

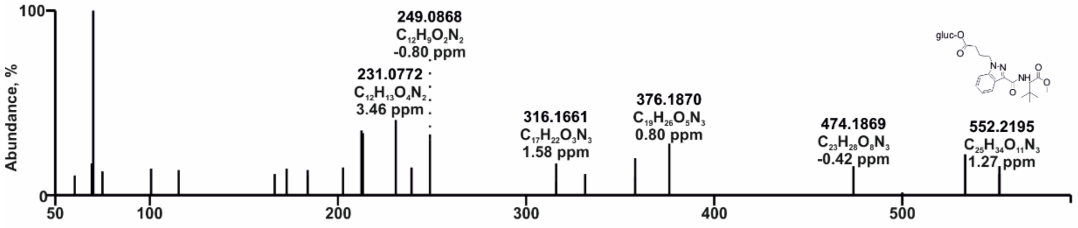

MB24 Oxidative defluorination + oxidation to carboxylic acid + glucuronidation, MS^2 at *m/z* 552.2188, RT 6.30 min

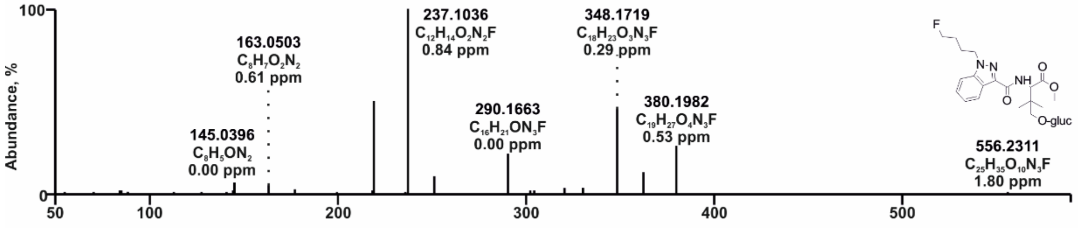

MB25 Hydroxylation of the t*ert*-butyl part + glucuronidation, MS^2 at *m/z* 556.2301, RT 6.35 min

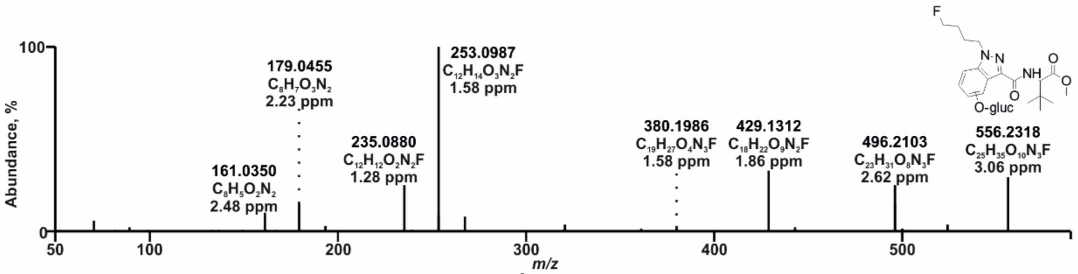

MB26 Hydroxylation of the indazole part + glucuronidation, MS^2 at *m/z* 556.2301, RT 6.58 min

Figure S1 (continued)

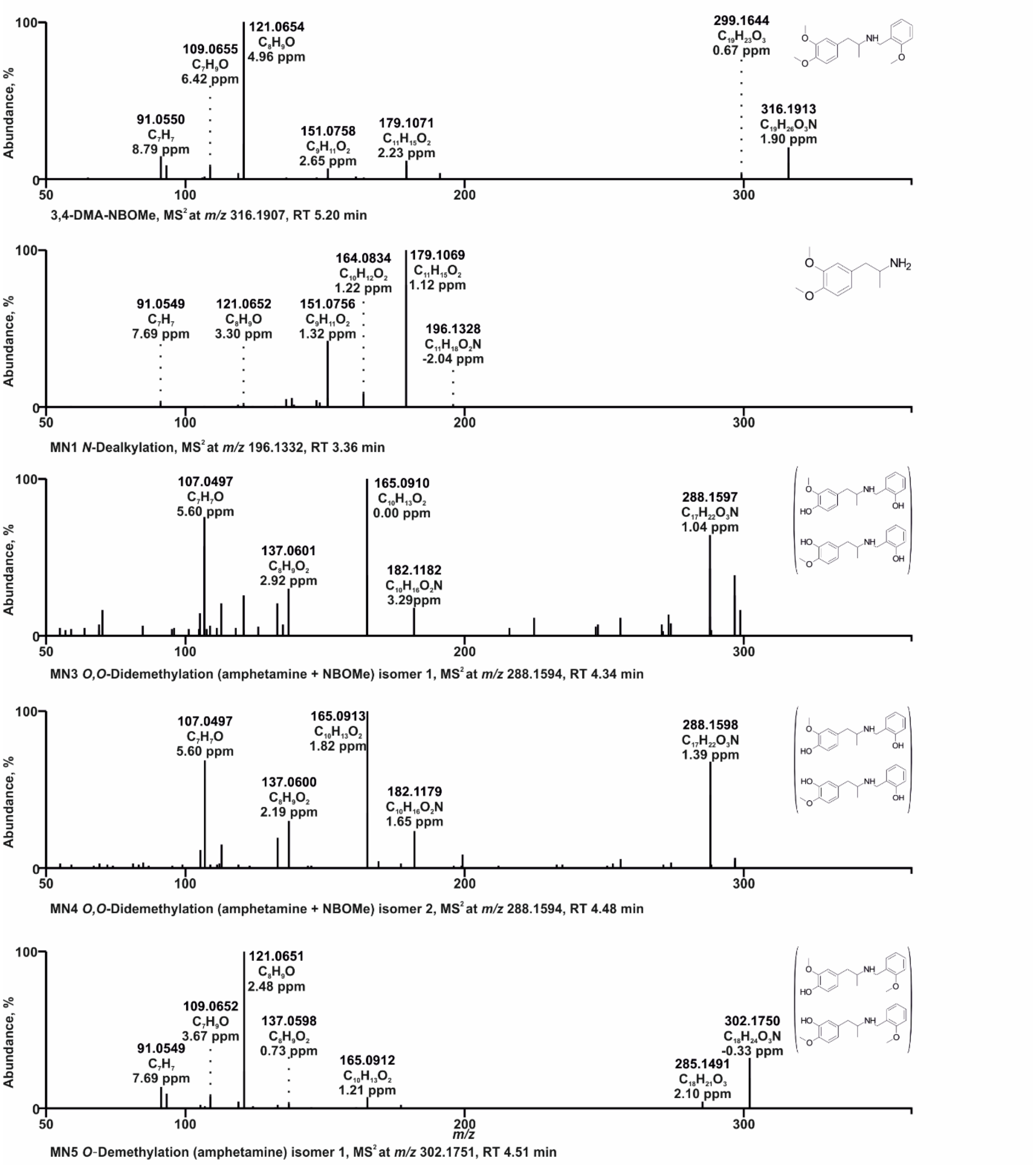

Figure S2 HRMS[2] spectra of 3,4−DMA−NBOMe and its metabolites detected in incubations with HepaRG cells and/or zebrafish larvae. MN, 3,4−DMA−NBOMe metabolite; RT, retention time

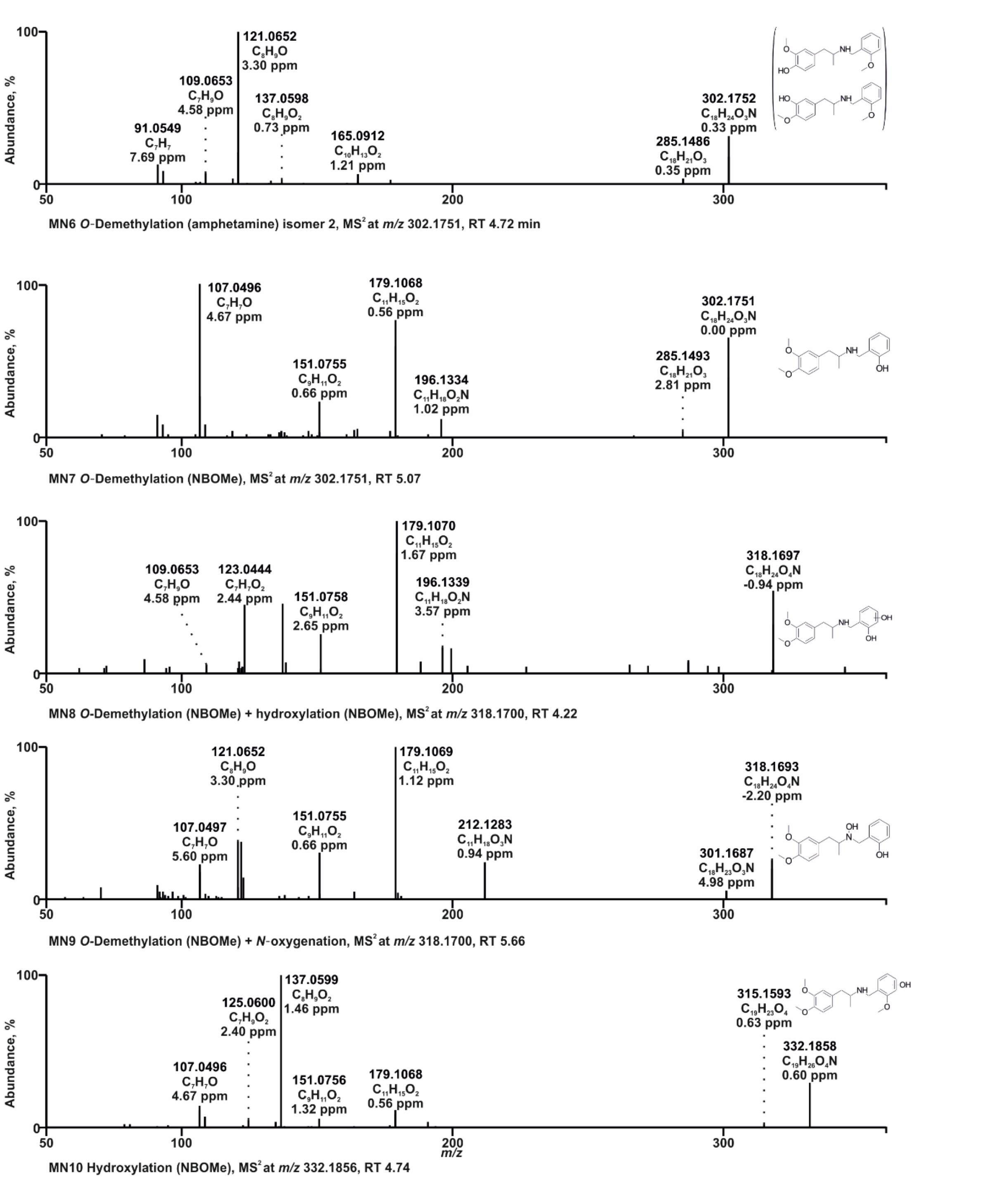

Figure S2 (continued)

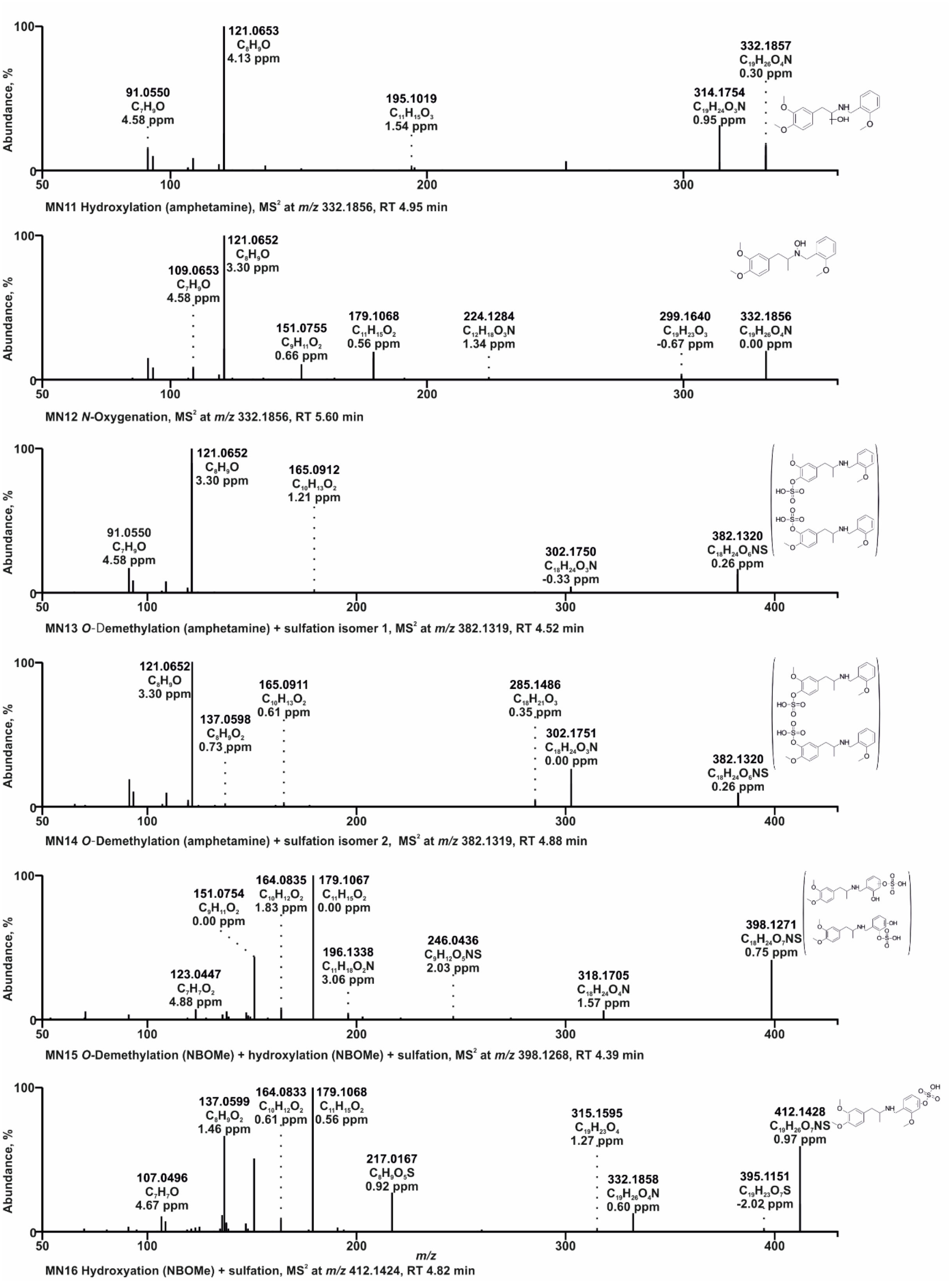

Figure S2 (continued)

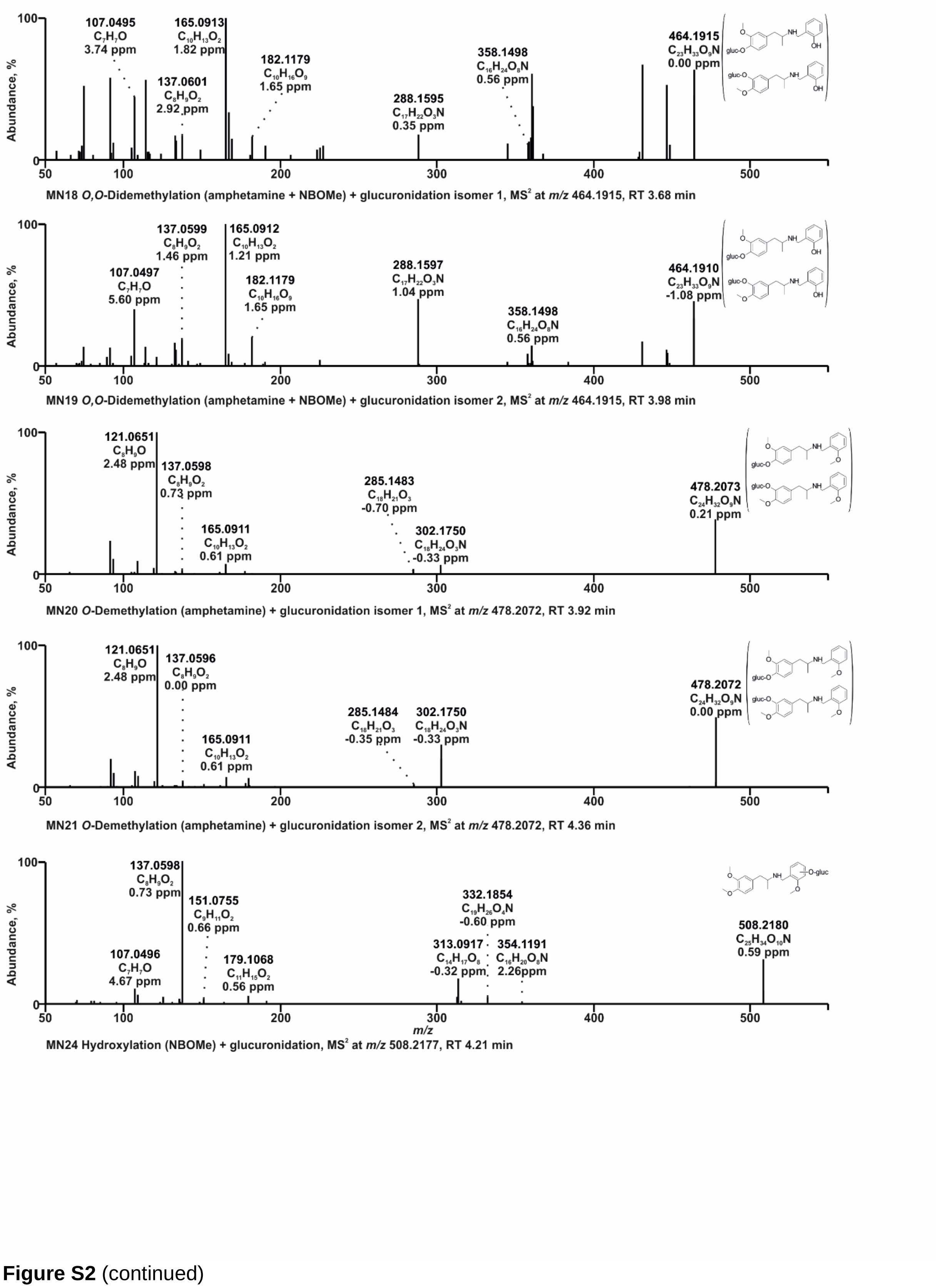

Figure S2 (continued)

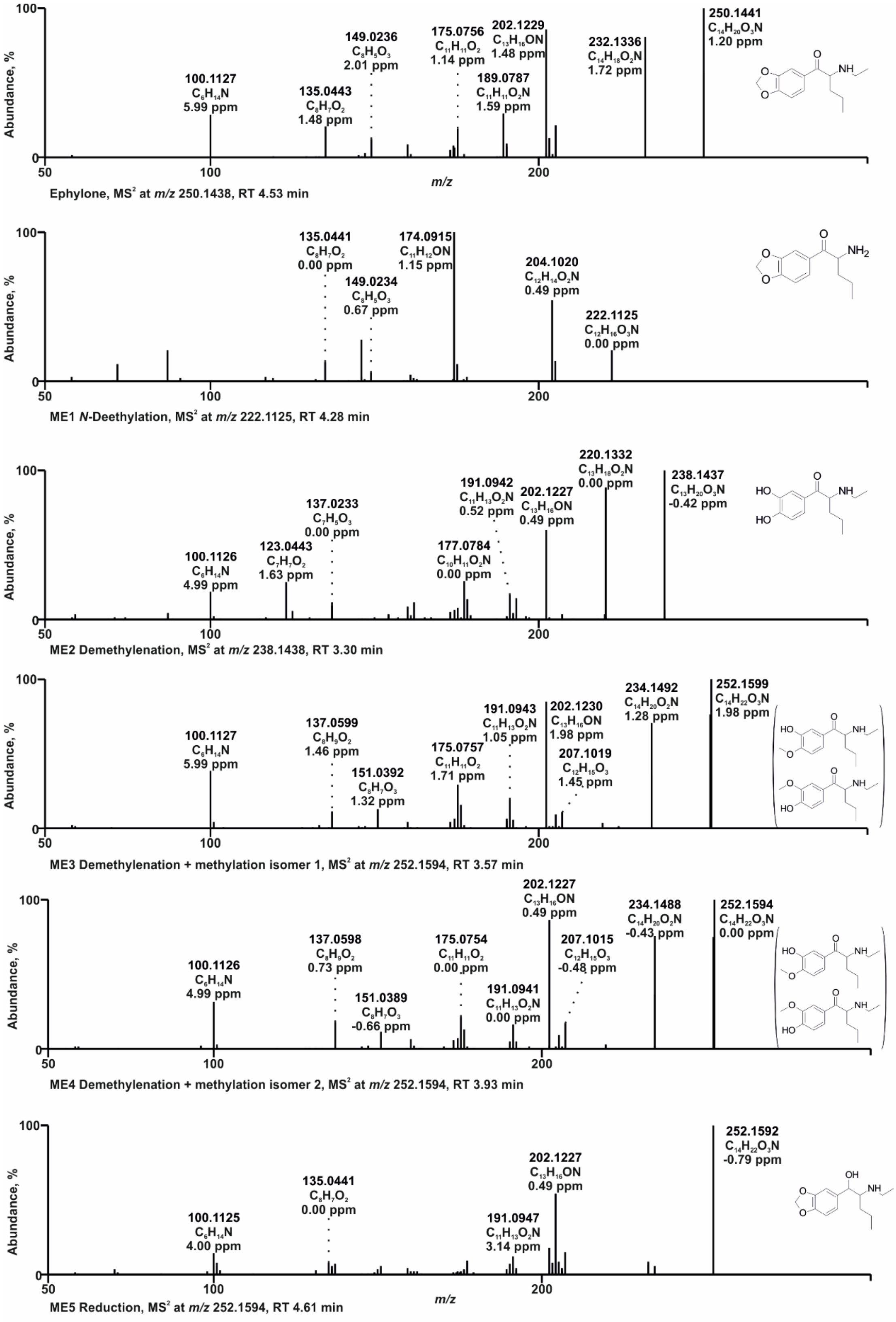

Figure S3 HRMS[2] spectra of ephylone and its metabolites detected in incubations with HepaRG cells and/or zebrafish larvae. ME, ephylone metabolite; RT, retention time

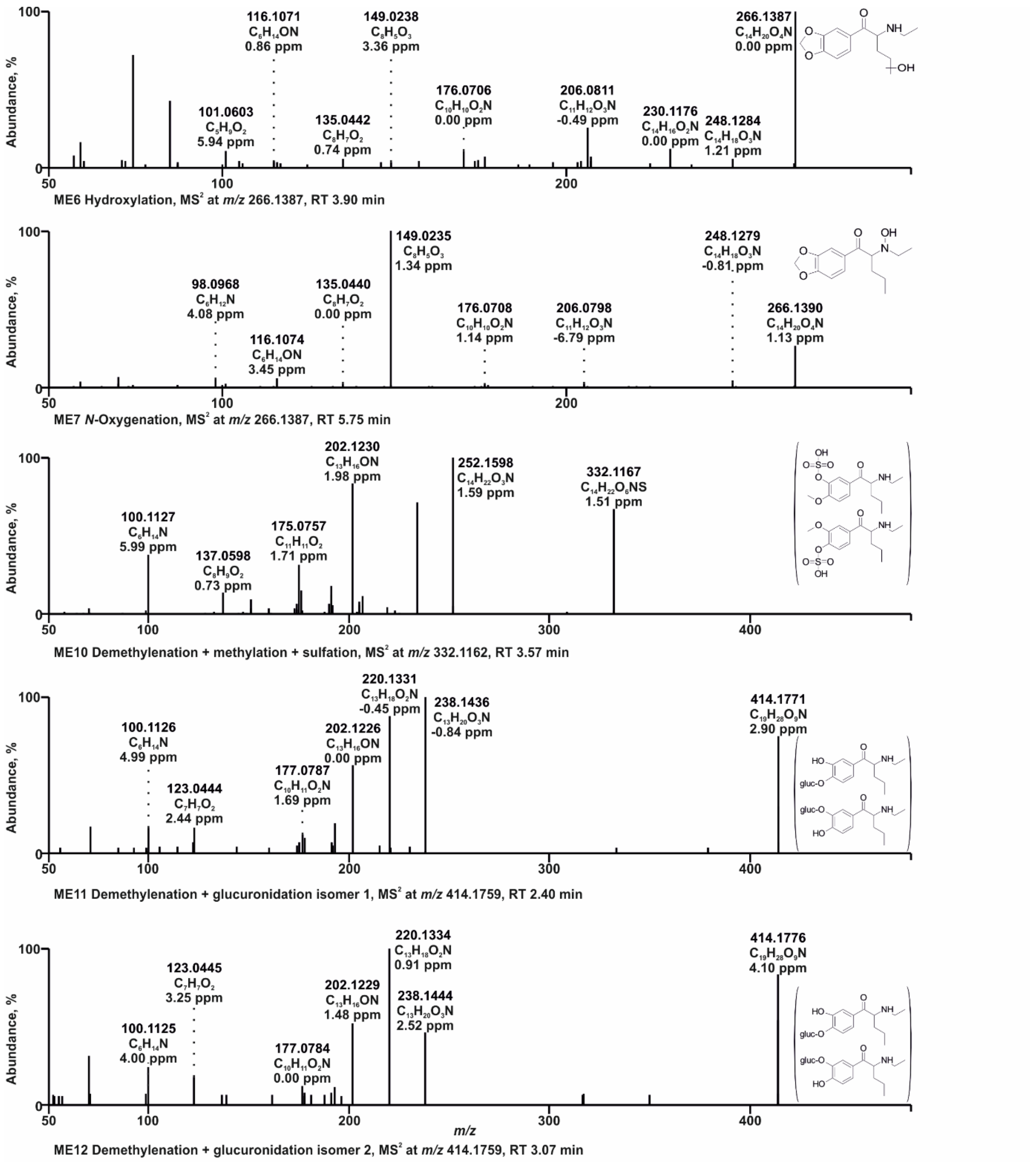

Figure S3 (continued)

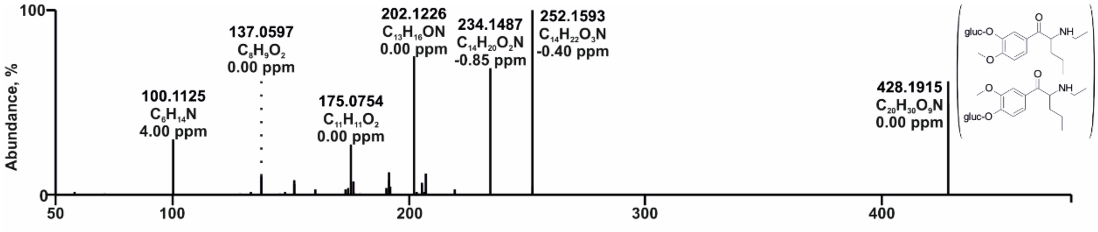

ME13 Demethylenation + methylation + glucuronidation isomer 1, MS^2 at *m/z* 428.1915, RT 2.87 min

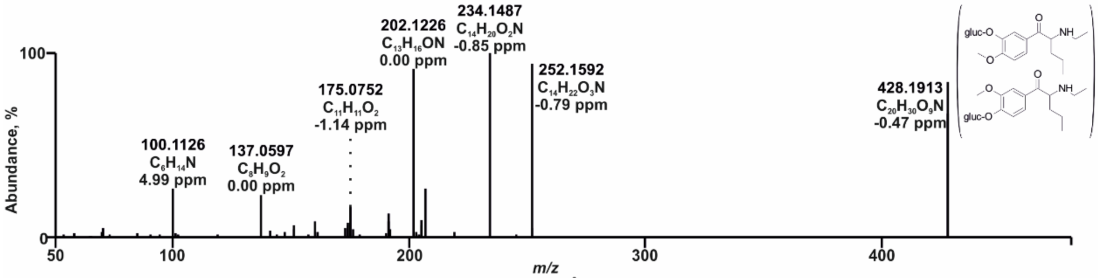

ME14 Demethylenation + methylation + glucuronidation isomer 2, MS^2 at *m/z* 428.1915, RT 3.29 min

Figure S3 (continued)

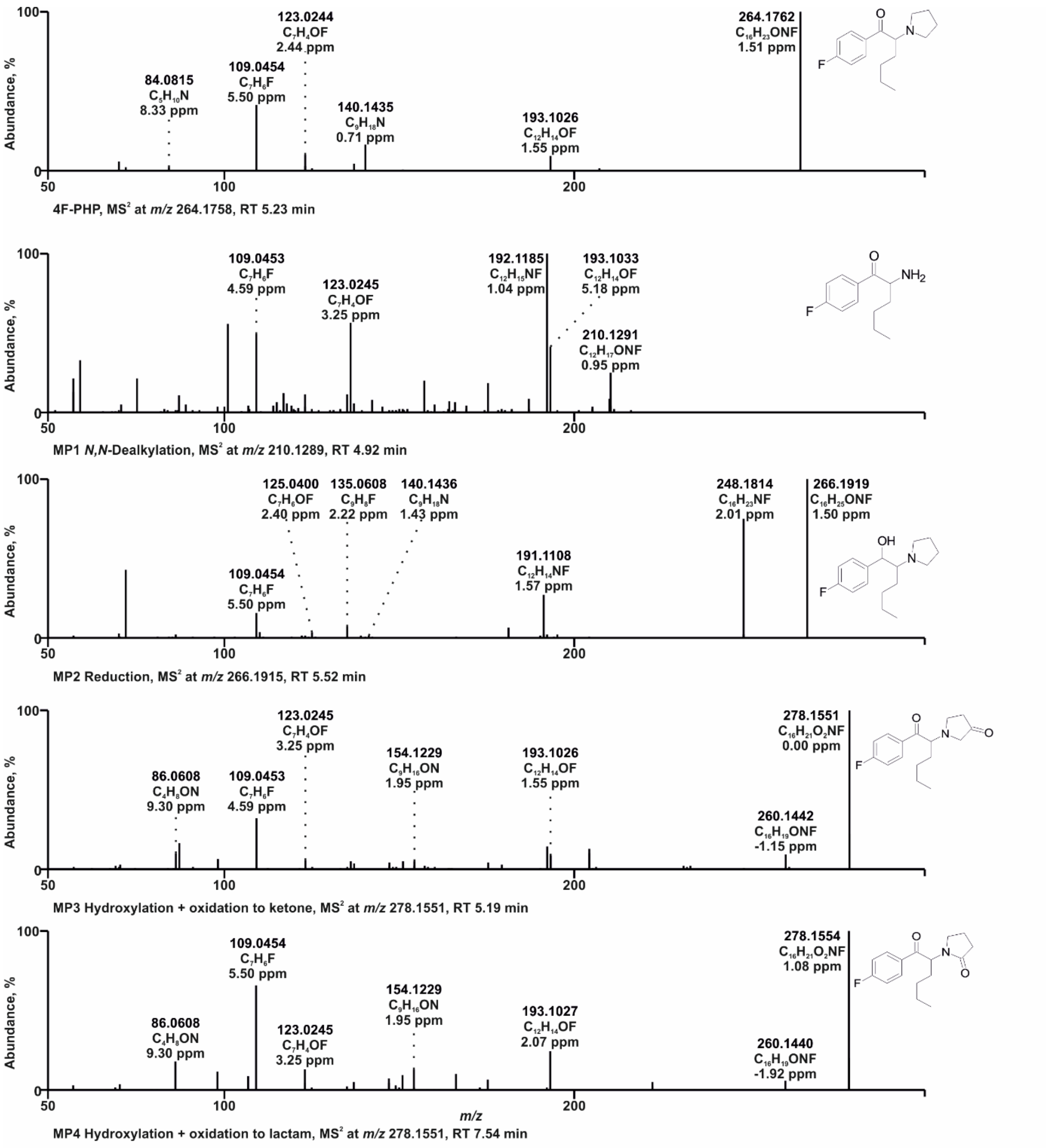

Figure S4 $HRMS^2$ spectra of 4F−PHP and its metabolites detected in incubations with HepaRG cells and/or zebrafish larvae. MP, 4F−PHP metabolite; RT, retention time

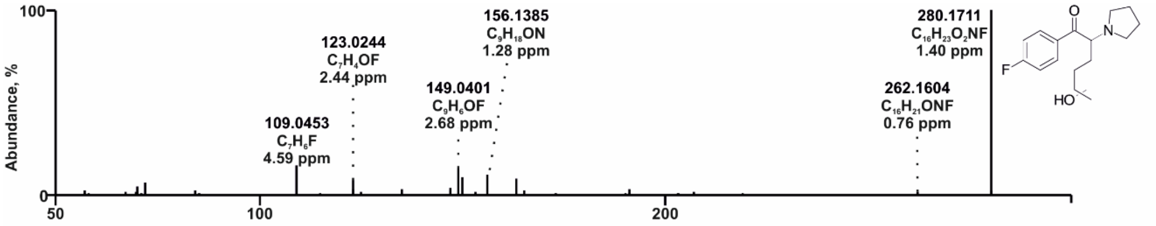

MP5 Hydroxylation isomer 1, MS[2] at *m/z* 280.1707, RT 3.86 min

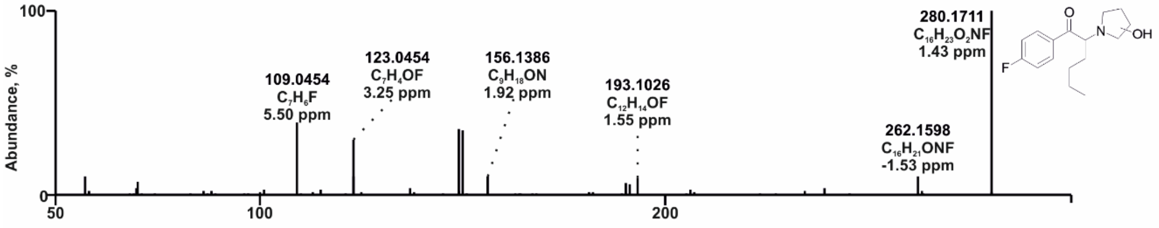

MP7 Hydroxylation isomer 3, MS[2] at *m/z* 280.1707, RT 5.02 min

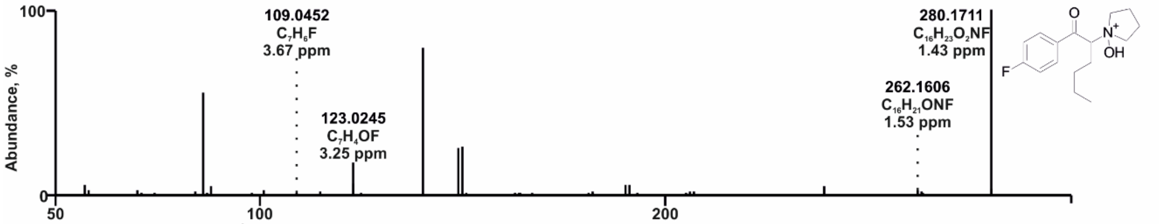

MP8 *N*-Oxygenation, MS[2] at *m/z* 280.1707, RT 5.78 min

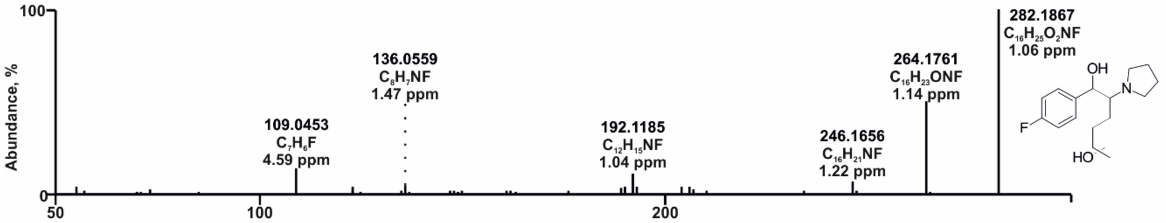

MP9 Reduction + hydroxylation, MS[2] at *m/z* 282.1864, RT 5.20 min

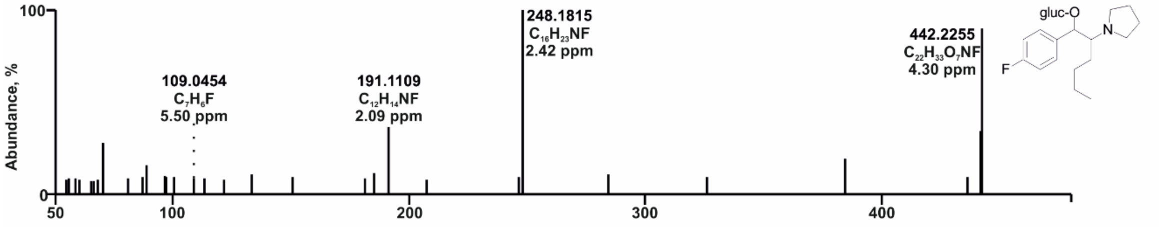

MP10 Reduction + glucuronidation, MS[2] at *m/z* 442.2236, RT 4.96 min

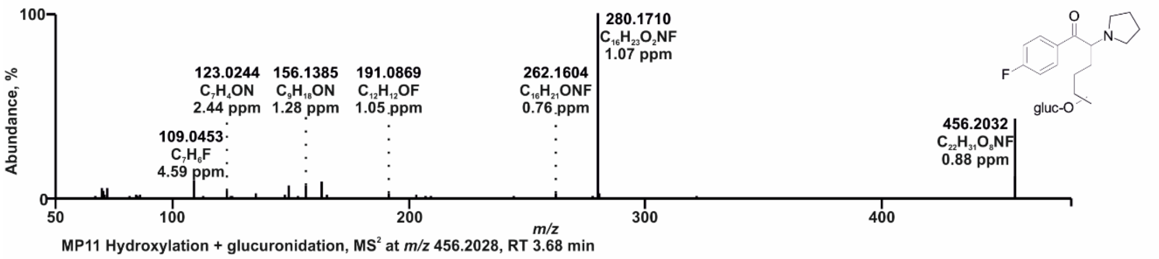

MP11 Hydroxylation + glucuronidation, MS[2] at *m/z* 456.2028, RT 3.68 min

Figure S4 (continued)

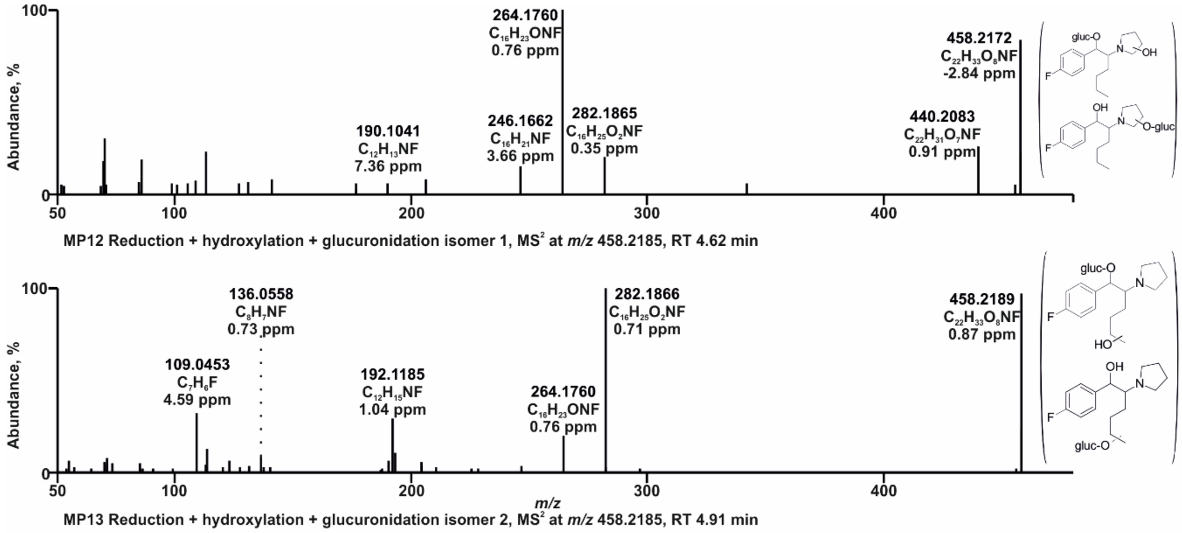

Figure S4 (continued)

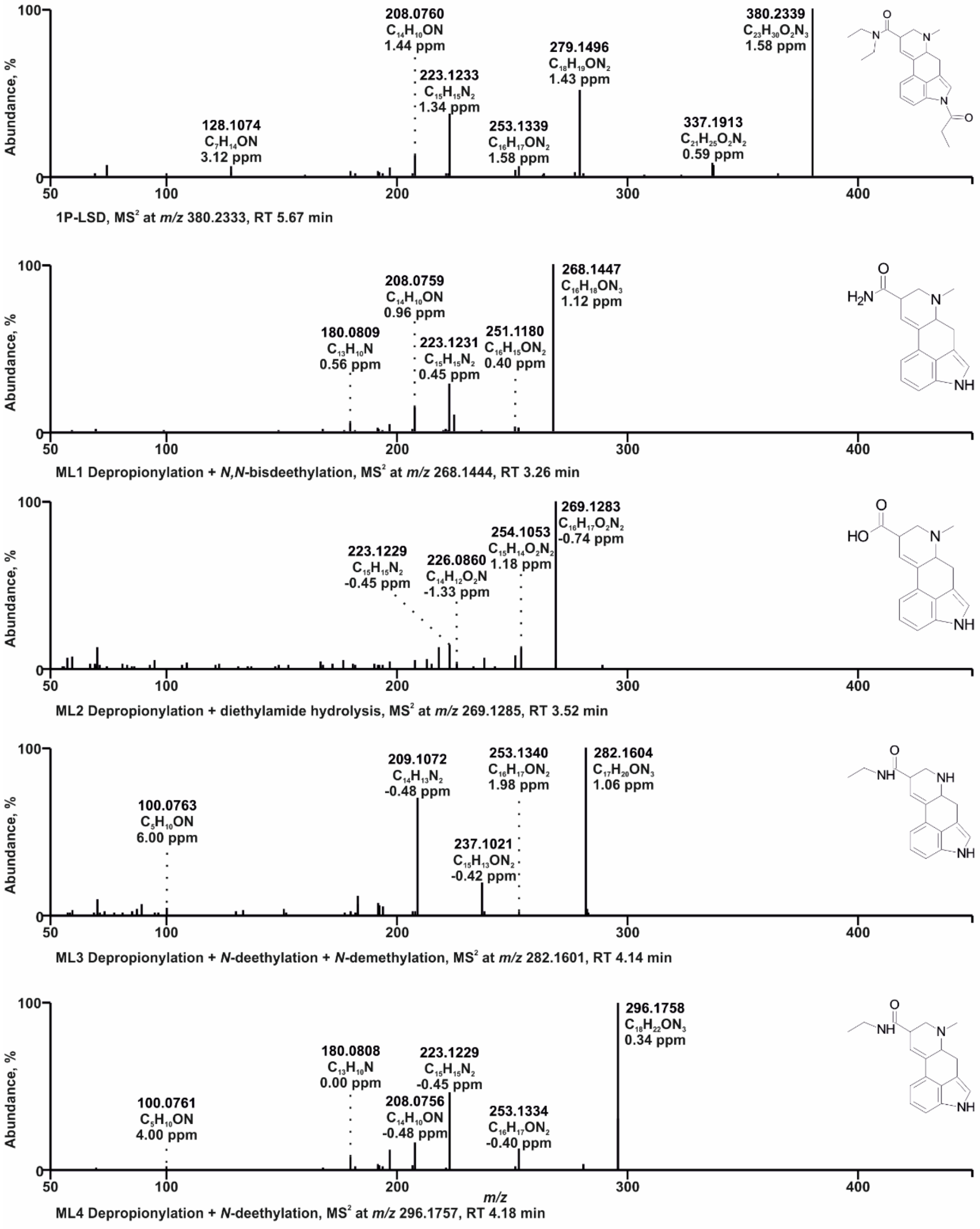

Figure S5 HRMS^2 spectra of 1P−LSD and its metabolites detected in incubations with HepaRG cells and/or zebrafish larvae. ML, 1P−LSD metabolite; RT, retention time

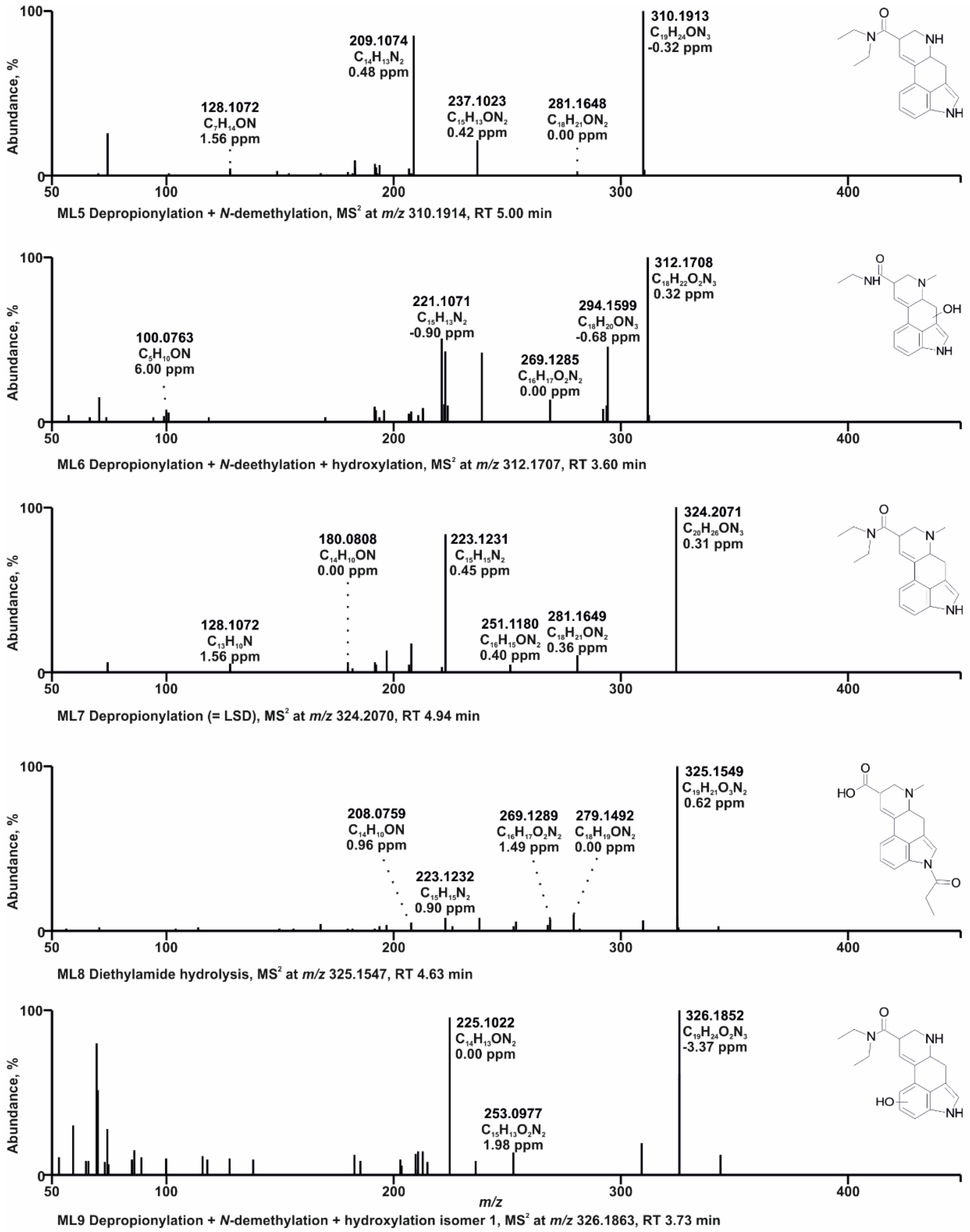

Figure S5 (continued)

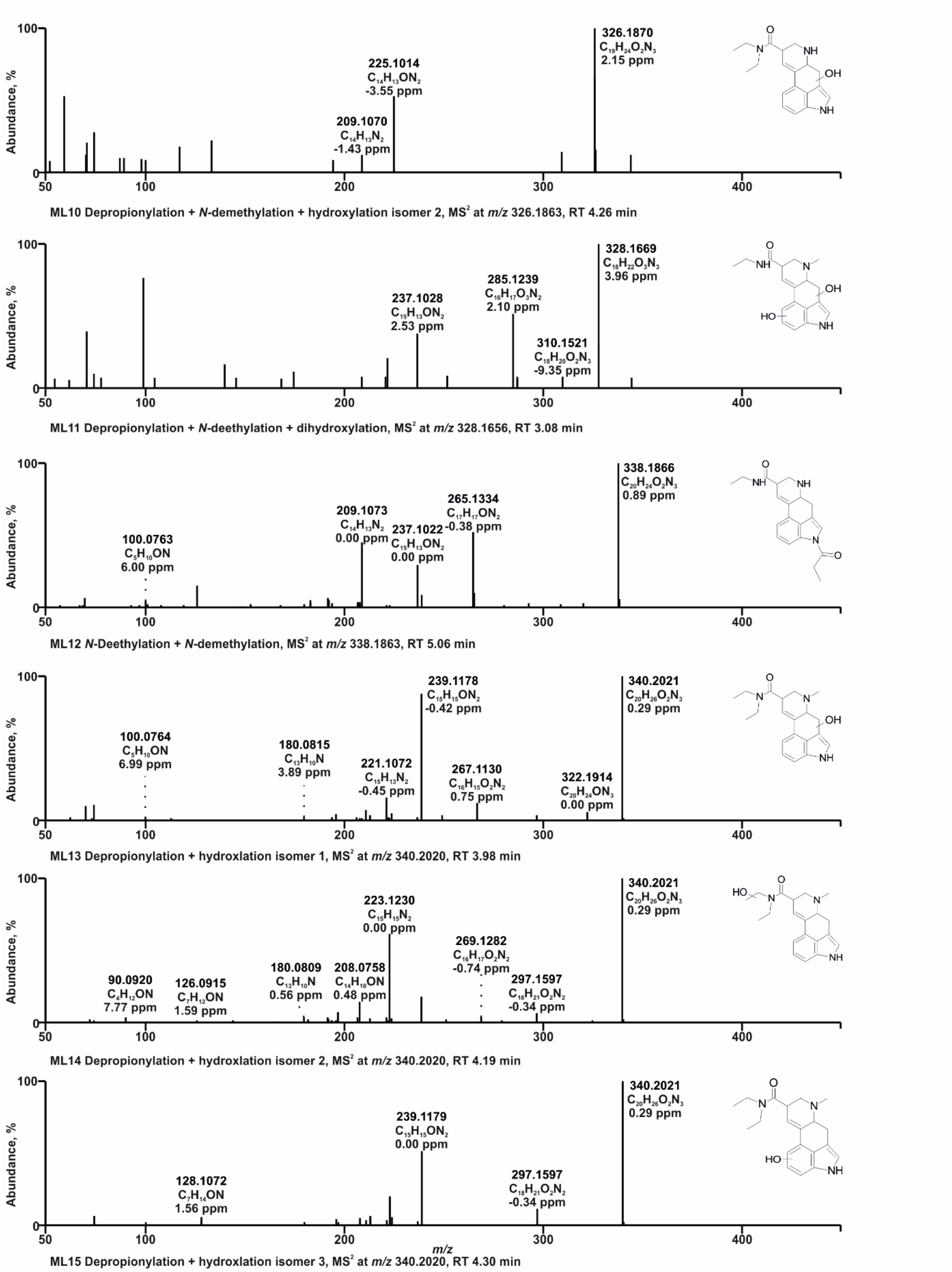

Figure S5 (continued)

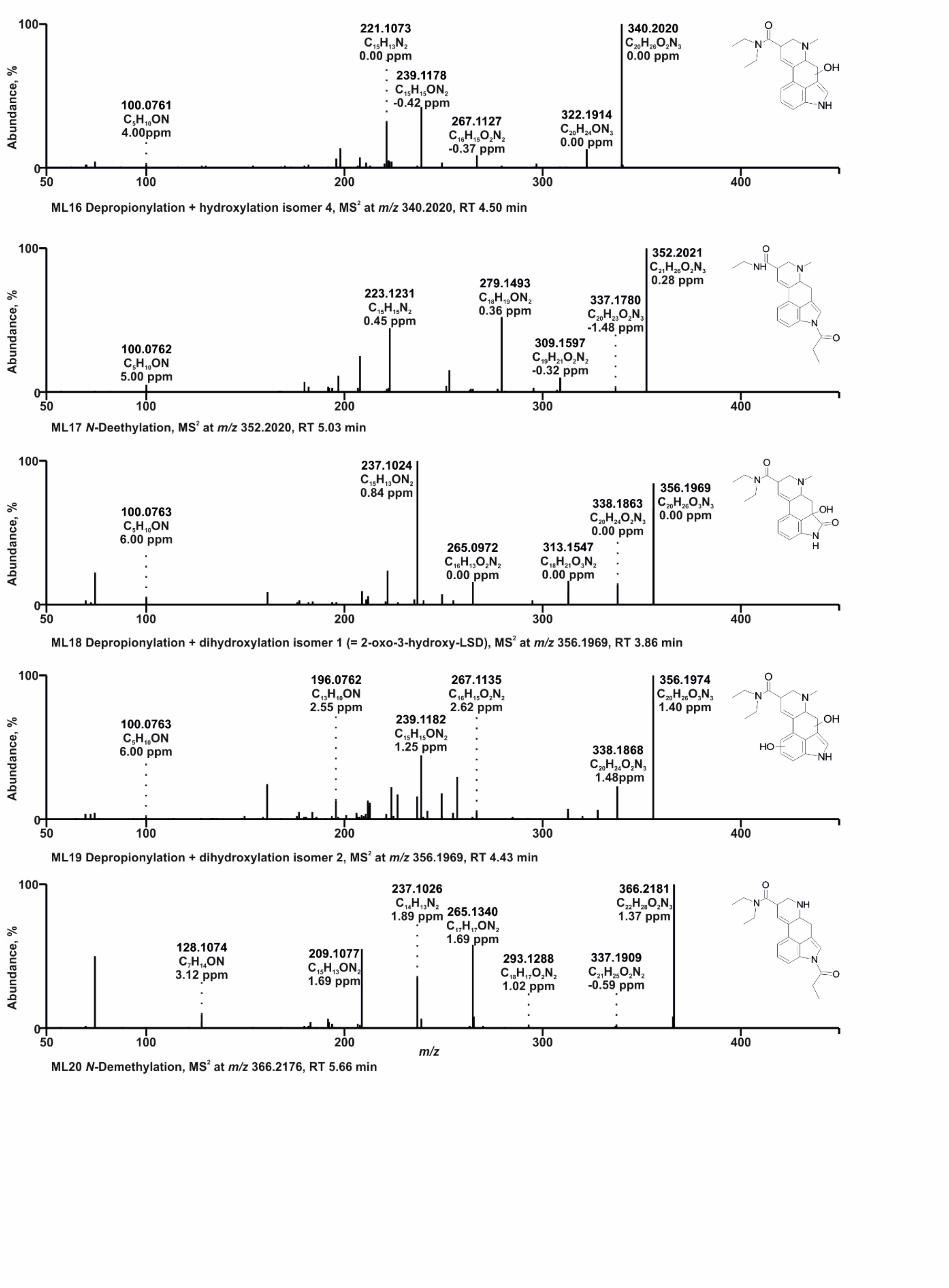

Figure S5 (continued)

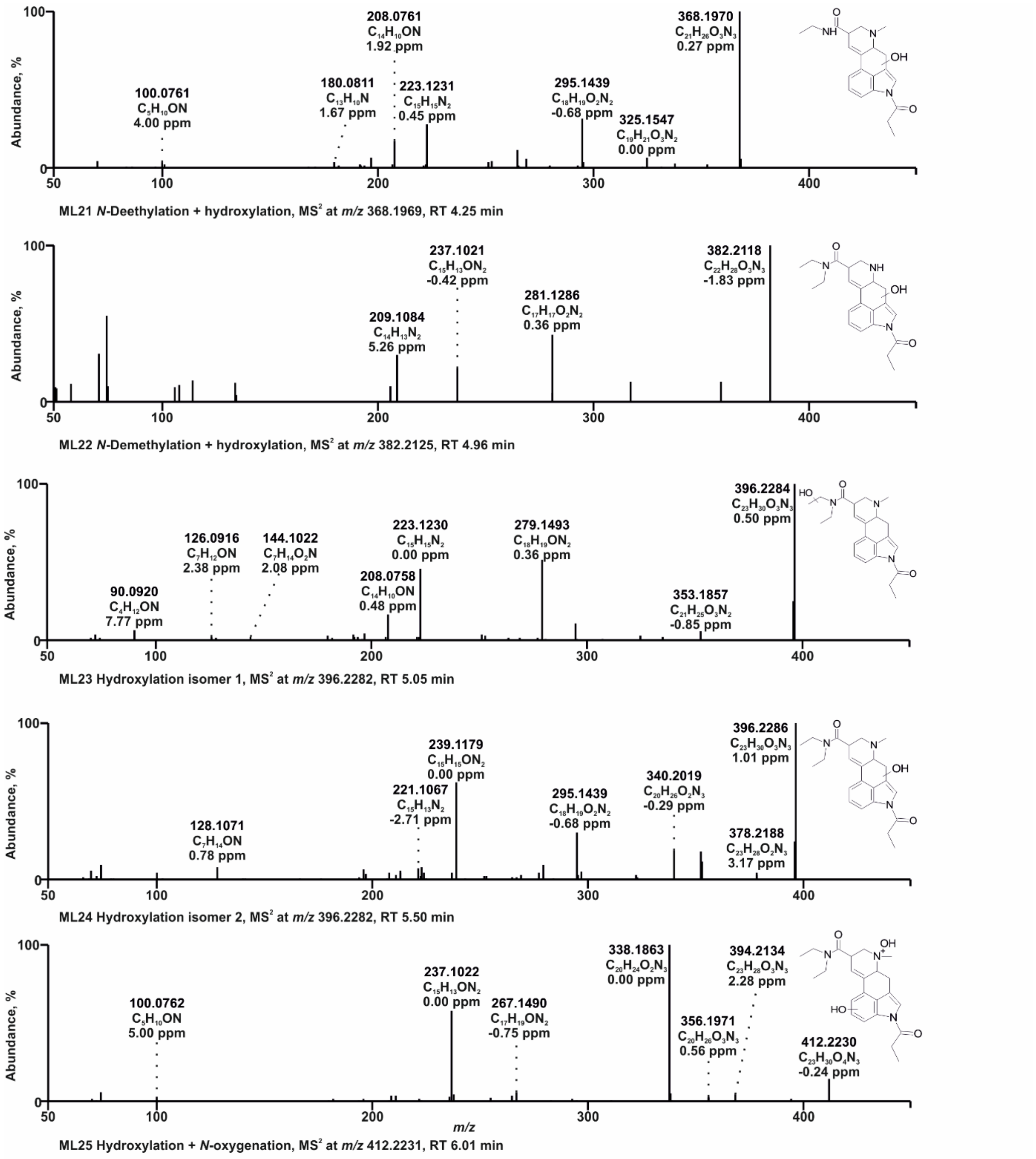

Figure S5 (continued)

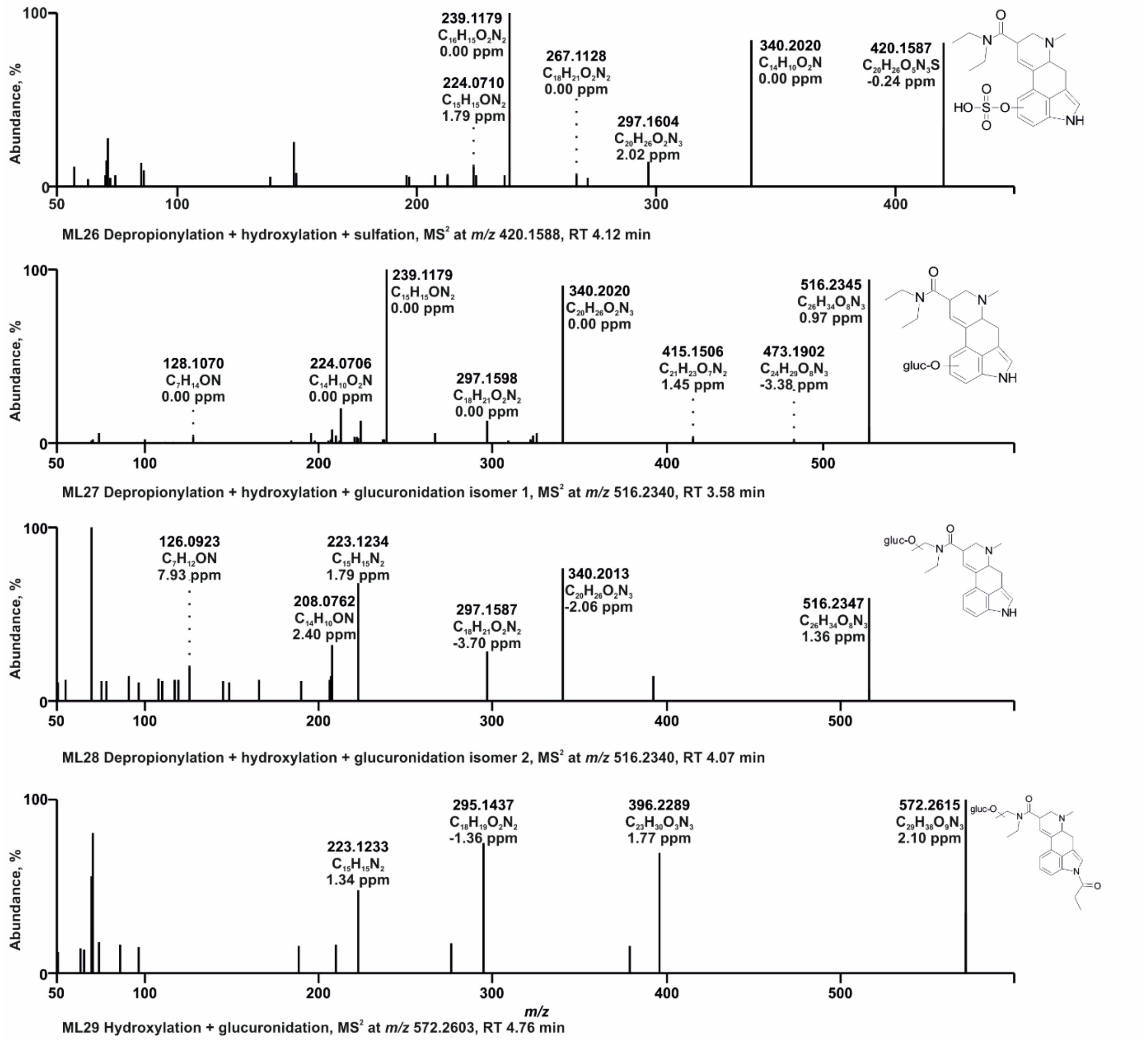

Figure S5 (continued)

REFERENCES

Caspar, A.T., Meyer, M.R., Westphal, F., Weber, A.A., and Maurer, H.H. (2018). Nano liquid chromatography−high−resolution mass spectrometry for the identification of metabolites of the two new psychoactive substances N−(ortho−methoxybenzyl)−3,4−dimethoxyamphetamine and N−(ortho−methoxybenzyl)−4−methylmethamphetamine. *Talanta* 188**,** 111−123. doi: 10.1016/j.talanta.2018.05.064.

Wagmann, L., Manier, S.K., Eckstein, N., Maurer, H.H., and Meyer, M.R. (2019a). Toxicokinetic studies of the four new psychoactive substances 4−chloroethcathinone, N−ethylnorpentylone, N−ethylhexedrone, and 4−fluoro−alpha−pyrrolidinohexiophenone. *Forensic Toxicology* in press. doi: 10.1007/s11419−019−00487−w.

Wagmann, L., Richter, L.H.J., Kehl, T., Wack, F., Bergstrand, M.P., Brandt, S.D., et al. (2019b). In vitro metabolic fate of nine LSD−based new psychoactive substances and their analytical detectability in different urinary screening procedures. *Anal Bioanal Chem* 411(19)**,** 4751−4763. doi: 10.1007/s00216−018−1558−9.
